# Costimulatory Effect of Rough Calcium Phosphate Coating and Blood Mononuclear Cells on Adipose-Derived Mesenchymal Stem Cells In Vitro as a Model of In Vivo Tissue Repair

**DOI:** 10.3390/ma13194398

**Published:** 2020-10-02

**Authors:** Igor A. Khlusov, Larisa S. Litvinova, Valeria V. Shupletsova, Olga G. Khaziakhmatova, Vladimir V. Malashchenko, Kristina A. Yurova, Egor O. Shunkin, Vasilii V. Krivosheev, Ekaterina D. Porokhova, Anastasiia E. Sizikova, Linara A. Safiullina, Elena V. Legostaeva, Ekaterina G. Komarova, Yurii P. Sharkeev

**Affiliations:** 1 Center for Immunology and Cell Biotechnology, Immanuel Kant Baltic Federal University, 236029 Kaliningrad, Russia; vshupletsova@mail.ru (V.V.S.); hazik36@mail.ru (O.G.K.); vlmalashchenko@kantiana.ru (V.V.M.); kristina_kofanova@mail.ru (K.A.Y.); egor.shunkin@gmail.com (E.O.S.); v_krivosheev@inbox.ru (V.V.K.); 2 Research School of Chemistry and Applied Biomedical Sciences, National Research Tomsk Polytechnic University, 634050 Tomsk, Russia; 3 Department of Morphology and General Pathology, Siberian State Medical University, 634050 Tomsk, Russia; porohova_e@mail.ru (E.D.P.); a.e.sizikova@gmail.com (A.E.S.); saflee4505@mail.ru (L.A.S.); 4 Laboratory of Physics of Nanostructured Biocomposites, Institute of Strength Physics and Materials Science SB RAS (ISPMS SB RAS), 634055 Tomsk, Russia; lego@ispms.tsc.ru (E.V.L.); katerina@ispms.ru (E.G.K.); sharkeev@ispms.tsc.ru (Y.P.S.); 5 Research School of High-Energy Physics, National Research Tomsk Polytechnic University, 634050 Tomsk, Russia

**Keywords:** microarc calcium phosphate coating, surface morphology, mice, subcutaneous behavior of implants, in vitro modeling, human cells, immunophenotype, motility, cytokine/chemokine secretion, osteogenic differentiation

## Abstract

Calcium phosphate (CaP) materials do not always induce ectopic vascularization and bone formation; the reasons remain unclear, and there are active discussions of potential roles for post-implantation hematoma, circulating immune and stem cells, and pericytes, but studies on adipose-derived stem cells (AMSCs) in this context are lacking. The rough (average surface roughness *R_a_* = 2–5 µm) scaffold-like CaP coating deposited on pure titanium plates by the microarc oxidation method was used to investigate its subcutaneous vascularization in CBA/CaLac mice and in vitro effect on cellular and molecular crosstalk between human blood mononuclear cells (hBMNCs) and AMSCs (hAMSCs). Postoperative hematoma development on the CaP surface lasting 1–3 weeks may play a key role in the microvessel elongation and invasion into the CaP relief at the end of the 3rd week of injury and BMNC migration required for enhanced wound healing in mice. Satisfactory osteogenic and chondrogenic differentiation but poor adipogenic differentiation of hAMSCs on the rough CaP surface were detected in vitro by differential cell staining. The fractions of CD73^+^ (62%), CD90^+^ (0.24%), and CD105^+^ (0.41%) BMNCs may be a source of autologous circulating stem/progenitor cells for the subcutis reparation, but allogenic hBMNC participation is mainly related to the effects of CD4^+^ T cells co-stimulated with CaP coating on the in vitro recruitment of hAMSCs, their secretion of angiogenic and osteomodulatory molecules, and the increase in osteogenic features within the period of in vivo vascularization. Cellular and molecular crosstalk between BMNCs and AMSCs is a model of effective subcutis repair. Rough CaP surface enhanced angio- and osteogenic signaling between cells. We believe that preconditioning and/or co-transplantation of hAMSCs with hBMNCs may broaden their potential in applications related to post-implantation tissue repair and bone bioengineering caused by microarc CaP coating.

## 1. Introduction

Currently, experimentally induced ectopic test is a well-studied, well-described model that is most commonly generated by subcutaneous, intramuscular, or kidney capsule transplantation. All 3 model types are valid experimental approaches to study the osteogenic differentiation of mesenchymal stem cells (MSCs) independent of an osseous environment [1], as well as the reconstitution of bone marrow organization, and to produce valuable information on the relations between hematopoietic cells and their microenvironment [2]. In vivo results allow us to estimate key molecular, cellular and tissue targets and strategies for regenerative medicine; in addition, such data improve our understanding of the biology of heterotopic calcification and ossification and the complications of implants for traumatology and orthopedics.

Despite continuous investigations of ectopic osteogenesis (EO) since at least 1952 [3], the full potential of bone marrow remodeling in ectopic implants has not yet been fully realized. Ectopically implanted marrow undergoes a regenerative process that recapitulates marrow ontogeny; this process is possible because marrow tissue has considerable angiogenic potential [2] and is a source of MSCs.

Bone marrow-derived MSCs (BM-MSCs) and adipose-derived MSCs (AMSCs) are commonly used in skeletal tissue engineering in vitro and in vivo [4]. Overall, BM-MSCs are more prone to osteogenic differentiation than AMSCs and show superior ectopic bone formation without the need for additional growth factors [5].

An experimental approach to evaluating remodeling of the bone/marrow system in the presence of artificial materials is a variant of EO when artificial samples have been implanted subcutaneously with bone marrow [6], BM-MSCs, progenitor cells [7], or other cells [8] without additional growth factors, mainly bone morphogenic proteins (BMPs). At the same time, an incidence of EO varied from 67 to 100%, while it was triggered by different types of microarc calcium phosphate (CaP) coatings on a titanium substrate [6].

Besides, inorganic biomaterials, mainly CaPs, can induce direct ectopic bone formation without the addition of osteogenic cells or bone growth factors when implanted under the skin or in muscle [1,9,10,11]; however, the underlying mechanisms remain unclear. At the same time, there have been a few reports stating that CaP scaffolds without cells did not show new bone formation at 8 weeks after subcutaneous implantation [12].

The formation of new blood vessels from pre-existing blood vessels (angiogenesis) is likely critical for ossification [13]. Skin injury results in the homing of blood progenitor cells [14] and leukocytes from the circulation to defects. Therefore, Scott et al., cannot exclude the participation of these progenitor cell types from ectopic bone formation [1]. On the other hand, pericytes (perivascular cells) that lie on the abluminal surface of blood microvessels and capillaries may be triggered or function as a source of activated MSCs that can differentiate into osteoblasts when in close contact with an osteogenic material [13]. Microparticles derived from CaP biomaterials (debris, degradation products, and commercial-grade particles) have been hypothesized to provoke inflammation and cytokine release. In some cases, it promotes the osteogenic differentiation of stem and progenitor cells [15]. Thus, pericytes and circulating stem/progenitor cells are possible candidates for osteoblast differentiation in the context of EO [16].

Poor subcutaneous vascularization and blood flow compared with muscle and kidney [1] obviously limit the participation of circulating MSCs and pericytes in subcutaneous osteoinduction on the implant surface and/or bulk before blood flow is restored. As a result, questions about the tissue origin of osteogenic cells (e.g., connective fat and skin) that can initiate angiogenesis and bone formation in such EO models remain unanswered.

AMSCs from subcutaneous fat that localize to the perivascular compartment promote local angiogenesis [17] and tissue formation as precursors of pericytes and other cells (adipocytes, osteoblasts, chondrocytes, endothelial cells, myocytes, etc.) [18] and secrete multiple cytokines and chemokines [17]. In turn, the invasion of blood mononuclear leukocytes into implant-dependent lesions leads to cooperation with local MSCs, which initiates controlled proliferation, inflammation and tissue repair [19].

In this regard, there is considerable interest in studying the subcutaneous vascularization of rough CaP material deposited by the microarc oxidation (MAO) technique and in vitro models of its effect on cellular and molecular crosstalk among blood mononuclear cells and AMSCs to evaluate cell motility, the secretion of angiogenic molecules, and osteogenic features; these data will provide a potential pathway of reparative regeneration after the implantation of inorganic biomaterials.

## 2. Materials and Methods

### 2.1. Substrate Preparation and Deposition of the CaP Coating

Commercially available pure titanium VT1-0 plates (99.58 Ti, 0.12 O, 0.18 Fe, 0.07 C, 0.04 N, and 0.01 H, wt.%; 10 × 10 × 1 mm^3^) were used as substrates. The titanium plates were polished with a series of increasingly fine abrasive paper up to P1200, ultrasonically cleaned in distilled water and ethanol for 10 min, and dried in air. The CaP coating was deposited by the microarc oxidation (MAO) method using the Microarc 3.0 system (ISPMS SB RAS, Tomsk, Russia) with a DC pulsed power supply in the anodic regime as described previously [20,21]. The titanium specimen and titanium electrolytic bath served as the anode and cathode, respectively. The electrolyte suspension contained 20 wt.% aqueous solution of phosphoric acid, 6 wt.% dissolved hydroxyapatite (HA, Ca_10_(PO_4_)_6_(OH)_2_) powder, and 9 wt.% dissolved calcium carbonate (CaCO_3_). The MAO parameters for the bilateral deposition of the CaP coating on titanium substrates were as follows: pulse frequency, 50 Hz; pulse duration, 100 µs; deposition time, of 7–10 min; and electrical voltage, 150–200–250 V. Rough CaP surfaces were prepared with average surface roughness (*R_a_*) values in the range of 2.0–2.9, 3.0–3.9, and 4.0–4.9 µm.

Roughness amplitude parameters and the mean value of the profile element width within a sampling length were measured with a Talysurf 5–120 profilometer (Taylor Hobson Ltd., Leicester, UK). The linear distance between surface features (*S_m_*) as the width of surface peaks and valleys, the *R_a_* as an arithmetic mean of the absolute ordinate values within a sampling length and the peak-to-valley roughness (*R_z_*) were estimated.

The coating thickness on cross-sectional micrographs was determined with scanning electron microscopy (SEM; LEO EVO 50, Zeiss, Germany; Nanotech Center at ISPMS SB RAS, Tomsk, Russia). The surface morphology and topography of the coating were analyzed via SEM (Philips SEM 515, Amsterdam, The Netherlands) at Tomsk Materials Science Center for Collective Use (Tomsk, Russia). The surface area was randomly examined at 300 to 5000 × magnification. An Olympus GX-71 inverted metallographic microscope (Olympus Corporation, Tokyo, Japan) equipped with an Olympus DP 70 digital camera (Olympus Corporation, Tokyo, Japan) was used to obtain images of the coating and to locate attached cells and tissues.

The specimens before and after coating were balanced on a digital microanalytical balance (GR-202, A&D Company, Tokyo, Japan), and the bilateral coating mass was calculated.

Before biological testing, the samples were dry heat sterilized in a Binder FD53 oven (Binder GmbH, Tuttlingen, Germany) at 453 K for 1 h. The samples were usually placed in the bottom of a well in a 12-well plastic plate, except for the Cell-IQ experiment, in which the samples were attached vertically at one edge of the well with a clip.

### 2.2. In Vivo Implantation of CaP-Coated Substrates

Studies were performed with 10 male mice (CBA, 2 months old) in compliance with the principles of humane treatment of laboratory animals (approval no. 1923 on 28 March 2011; Local Ethics Committee, Siberian State Medical University, Tomsk, Russia; approval no. 7 on 9 December 9 2015; Local Ethics Committee of Innovation Park, Immanuel Kant Baltic Federal University, Kaliningrad, Russia). Mice were received from the Mouse Bank of Goldberg Research Institute of Pharmacology and Regenerative Medicine, Tomsk National Research Medical Center of the Russian Academy of Sciences, Tomsk, Russia; upon receipt, the mice were housed in the Central Scientific-Research Laboratory of Siberian State Medical University (annual veterinary certificate and compliance audit report). The animals were acclimatized to laboratory conditions (22 °C, 12 h/12 h light/dark, 50% humidity, ad libitum access to food and water) for 2 weeks prior to operation.

Mice were anesthetized with an intramuscular injection of Zoletil (5 mg/kg) (Vibrac Sante Animale, Carros, France) and Rometar (4 mg/kg) (Bioveta, Ivanovice na Hane, Czech Republik) and operated under sterile conditions. The skin was sterilized with 70% ethanol, twenty CaP-coated titanium samples were implanted into the lateral subcutis pockets of the venter, and the wound was sutured and treated with 70% ethanol.

Each week for 5 weeks, two animals were euthanized by CO_2_ inhalation. Two samples from each mouse were explanted and fixed for 24 h with neutral formalin. Then, the sample surface was studied with an Olympus GX-71 inverted metallographic microscope (Olympus Corporation, Tokyo, Japan) equipped with an Olympus DP 70 digital camera (Olympus Corporation, Tokyo, Japan). Some tissues grown on CaP surfaces were stained with hematoxylin and eosin.

### 2.3. Human Cell Isolation

Adult human AMSCs (hAMSCs) were isolated from lipoaspirates of a healthy man (29 years old) who was undergoing liposuction for aesthetic reasons in the surgery hospital. This study was approved by the Local Ethics Committee of Innovation Park, Immanuel Kant Baltic Federal University, Kaliningrad, Russia (permission no. 7 on 9 December 2015). Informed consent for the procedure was obtained as specified previously [22]. A stromal vascular fraction and a processed lipoaspirate (PLA) with little contamination by endothelial cells, pericytes, and smooth muscle cells were obtained as described previously [23].

PLA cells were passaged at subconfluence four times (each passage lasting 5–7 days) and cultured at 37 °C and 5% CO_2_ in nutrient medium consisting of 90% α-MEM (Sigma-Aldrich, St. Louis, MO, USA), 10% fetal bovine serum (Sigma-Aldrich, St. Louis, MO, USA), 0.3 g/L L-glutamine (Sigma-Aldrich, St. Louis, MO, USA), and 100 U/mL penicillin/streptomycin (Sigma-Aldrich, St. Louis, MO, USA) to expand the ex vivo hAMSC population. The cells were stained using a Human Phenotyping Kit (130-095-198) (Miltenyi Biotec, Bergisch-Gladbach, Germany) and Viability Fixable Dyes (Miltenyi Biotec, Bergisch-Gladbach, Germany), and the results were analyzed with a MACS Quant flow cytometer (Miltenyi Biotec, Bergisch-Gladbach, Germany) and KALUZA Analysis Software (Beckman Coulter, Brea, CA, USA) in accordance with the manufacturer’s instructions. As a result, most of the viable, adherent, fibroblast-like cells expressed CD73 (99%), CD90 (85%), and CD105 (99%) and did not show a hematopoietic immunophenotype (1% positive for CD45, CD34, CD20, and CD14).

Previously, PLA cells cultured with specific induction media from the StemPro^®^ Differentiation Kit (Thermo Fisher Scientific, Waltham, MA, USA) for 21 days showed multilineage differentiation into osteoblasts, chondrocytes, and adipocytes by selective staining [24]. Thus, the isolated cells constitute a pool of hAMSCs according to the recommendations of the International Society for Cellular Therapy (ISCT) and the International Federation for Adipose Therapeutics and Science (IFATS) [25,26].

Human blood mononuclear cells (hBMNCs) were collected from 3 young (25–32 years old) healthy men (permission no. 2 on 6 March 2017; Local Ethics Committee, Innovation Park, Immanuel Kant Baltic Federal University) by venous blood gradient (ρ = 1.077) Ficoll-Paque Premium (Sigma-Aldrich, St. Louis, MO, USA) centrifugation at 1500 rpm for 45 min. Informed consent was obtained from each volunteer for the diagnostic procedure. The hBMNCs were washed twice with phosphate-buffered saline (pH = 7.2) and resuspended in complete culture medium consisting of 90% α-MEM (Sigma-Aldrich, St. Louis, MO, USA), 10% inactivated (30 min at 56 °C) fetal bovine serum (Sigma-Aldrich, St. Louis, MO, USA), 0.3 g/L L-glutamine (Sigma-Aldrich, St. Louis, MO, USA), and 100 U/mL penicillin/streptomycin (Sigma-Aldrich, St. Louis, MO, USA). The cells were 92–94% viable, as shown by 0.4% trypan blue staining before culture.

### 2.4. Human Cell Culture

To obtain 2-day and 3-day monocultures, hBMNCs were resuspended in complete culture medium consisting of 90% α-MEM (Sigma-Aldrich, St. Louis, MO, USA), 10% inactivated (30 min at 56 °C) fetal bovine serum (Sigma-Aldrich, St. Louis, MO, USA), 0.3 g/L L-glutamine (Sigma-Aldrich, St. Louis, MO, USA), and 100 U/mL penicillin/streptomycin (Sigma-Aldrich, St. Louis, MO, USA). One CaP-coated substrate was placed in each well of a 12-well flat-bottom plate (Orange Scientific, Braine-l’Alleud, Belgium). A cell suspension without a three-dimensional (3D) substrate was used as a 2D control. The cells were plated at 1 × 10^6^ live cells per 1.5 mL of nutrient medium and then incubated in a humidified atmosphere of 95% air and 5% CO_2_ at 37 °C.

To study 14-day cell viability, secretion and immunophenotype, hBMNCs were cultivated at a concentration of 1 × 10^6^ live cells per 1.5 mL of nutrient medium as described above.

**Figure 1:**
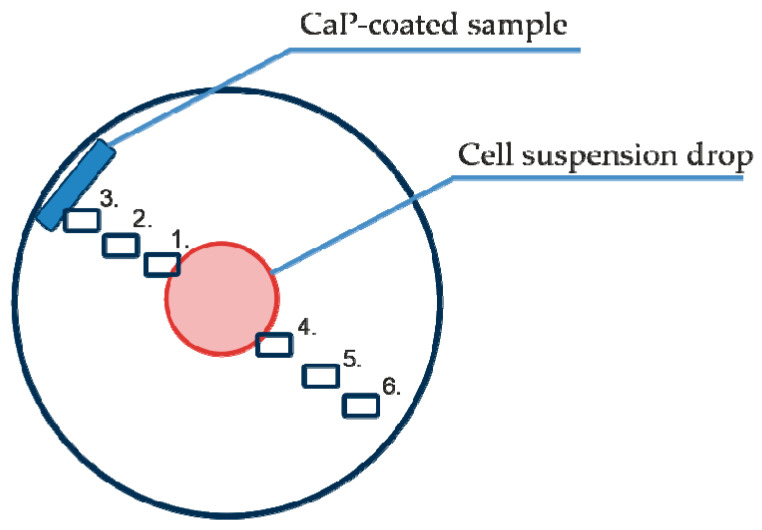
Scheme of cell migration imaging points 01–06 relative to the primary cell suspension and the test samples with calcium phosphate (CaP) coating.

For 7-day cell motility, hAMSCs or hBMNCs were resuspended in complete culture medium consisting of 90% α-MEM (Sigma-Aldrich, St. Louis, MO, USA), 10% inactivated (30 min at 56 °C) fetal bovine serum (Sigma-Aldrich, St. Louis, MO, USA), 0.3 g/L L-glutamine (Sigma-Aldrich, St. Louis, MO, USA), and 100 U/mL penicillin/streptomycin (Sigma-Aldrich, St. Louis, MO, USA). The nutrient medium was replaced with fresh medium every 3–4 days. One CaP-coated substrate was placed in each well of a 12-well flat-bottom plate (Orange Scientific, Braine-l’Alleud, Belgium). Samples were placed vertically in the well at one edge and attached to the wall with a clip. In this position, the samples did not shift when the plates were placed in the Cell-IQ^®^ v2 MLF integrated platform for continuous real-time phase-contrast imaging of live cells (CM Technologies Oy, Tampere, Finland) and did not damage the growing cell layer. Then, 50 μL of cell suspension (5 × 10^4^ viable hAMSCs or hBMNCs) was placed in the center of the well. Allogenic cell coculture (5 × 10^4^ viable hAMSCs and hBMNCs at a 1:1 ratio) was performed by mixing the cell suspensions at the volumes of 50 and 25 μL, respectively (Figure 1). Cell cultures without CaP-coated 3D substrates served as 2D controls. The cells were allowed to adhere to the bottom of the wells in a humidified chamber for 80 min. Thereafter, the wells were carefully filled with 1.5 mL of the nutrient medium, and the cells were observed in a Cell-IQ^®^ v2 MLF integrated platform for 7 days in a humidified atmosphere of 95% air and 5% CO_2_ at 37 °C until monolayer formation was detected. The nutrient medium was replaced with fresh medium in 3–4 days.

### 2.5. Cell-IQ Visualization of Cell Behavior

The Cell-IQ^®^ v2 MLF integrated platform (CM Technologies, Oy, Tampere, Finland) was used for continuous real-time phase-contrast imaging of live cells as described in our previous publication [27] with minor modifications. The monocultures or mixed cultures were placed into the wells of 12-well flat-bottom plates as described above and did not directly contact the CaP-coated samples for a long time, suggesting a predominantly indirect influence (from dissolution products of the coating material) of the CaP-coated specimens on cell behavior (Figure 1).

To analyze cell morphology, motility and division, six points were selected in each well for phase contrast imaging. The visualization fields were located as follows (Figure 1): on the cell layer boundary (1), equidistant between the cell layer and sample (2), near the sample (3), and equidistant between the cell drop and the sample but on the opposite side (4–6).

Digital microphotographs of the cell cultures (a total of 108 images for each of the six points) were obtained every 90 min. Several electronic libraries of digital images were created for effective cell identification. Every tenth image was used for automated analysis with Cell-IQ Imagen software (MI2.8.9, CM Technologies Oy, Tampere, Finland). The maximum cell amount and the number and timing of hAMSC divisions were determined at the chosen visualization points.

### 2.6. RTCA Technique to Monitor Cell Invasion and Recruitment

Cell migration through microholes (invasion) in the polymer membrane that imitate blood vessel pores was monitored with 16-well CIM plates for the real-time cell analyzer (RTCA) (xCELLigence RTCA DP system, Roche Applied Science, Penzberg, Germany). The system allows real-time determination of impedance dynamics at cell contacts with gold electrodes and of the cell index, which correlates with the number of cells and area of attachment to the electrode. Each well in the CIM plate is composed of two chambers: The lower chamber (maximum volume, 162 μL) contains chemoattractant, cells or tissues, and test cells are placed in the upper chamber (maximum volume, 180 μL). The cells migrate through 8 µm pores on the opposite surface of the membrane in the upper chamber, which is 80% covered with gold electrodes. The holder for the upper and lower chamber assembly was placed in a CO_2_ incubator for 24 h at 37 °С.

The experiment was performed according to previously described methods [28,29] with some modifications. All experiments were performed in laminar sterile air. Four wells were used for each experimental group. The hAMSCs or hBMNCs (4 × 10^4^ cells) in standard culture medium (α-MEM, 100 U/mL penicillin and streptomycin, and 0.3 g/L L-glutamine) were placed in the lower chamber. In the control wells, the lower chamber was filled with the corresponding volume (160 μL) of culture medium, the upper chamber was filled with 30 μL of culture medium, and the CIM plates were incubated for 20 min at 37 °C. CIM plates were placed in the RTCA DP Analyzer (Roche Applied Science, Penzberg, Germany) for instrument calibration. Then, the upper chambers of the CIM plate were filled with 4 × 10^4^ hBMNCs or hAMSCs in 150 μL of culture medium and placed in the RTCA DP Analyzer for 20 min at 37 °C. After that, the signals determining the migration index (MI) were recorded every 15 min for 72 h with the RTCA Software.

### 2.7. Cellular Immunophenotype and Viability Analysis

The cellular antigen profile was analyzed using specific monoclonal antibodies (mAbs; see below) according to the manufacturer’s instructions. The mAbs were labeled with fluorescein isothiocyanate (FITC), allophycocyanin (АРС), phycoerythrin (PE), or peridinin chlorophyll protein (PERCP).

**Figure 2:**
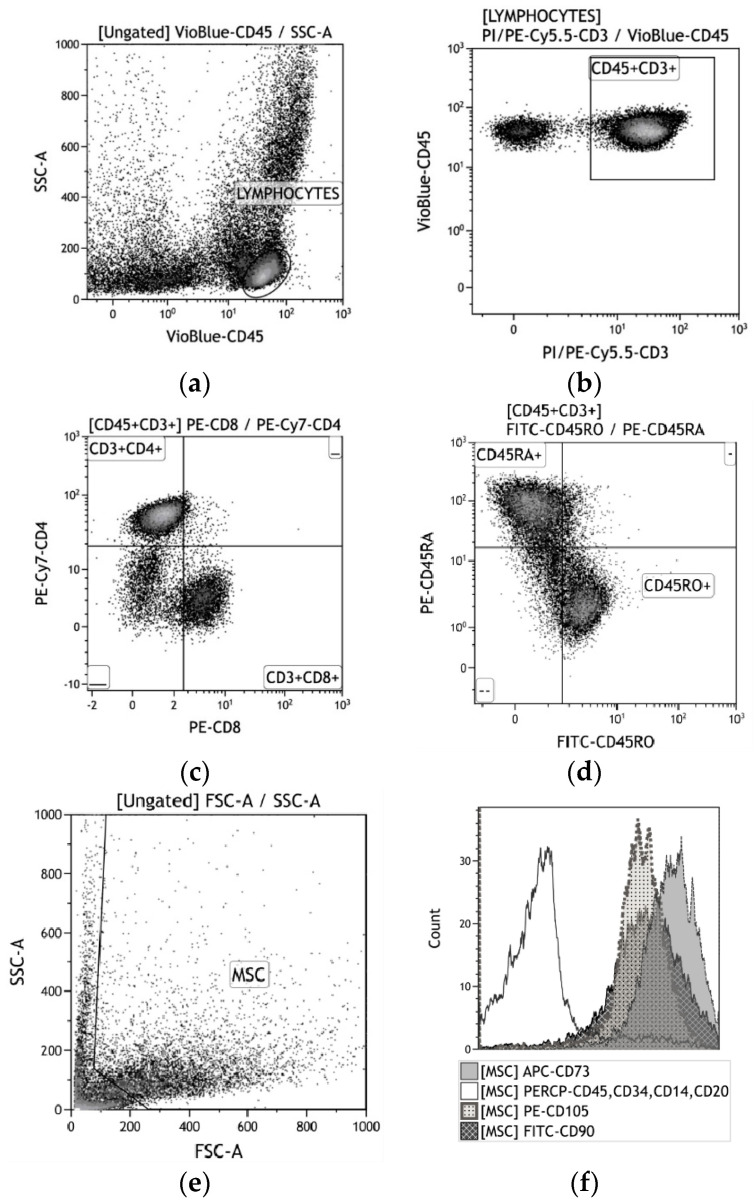
A strategy for gating live human blood mononuclear cells (hBMNC) subsets (**a**)–(**d**) and human adipose-derived stem cells (hAMSCs) (**e**)–(**f**). (**a**) CD45^+^ leukocytes; (**b**) CD45^+^CD3^+^ T cells; (**c**) CD4^+^ vs. CD8^+^ T cells; (**d**) CD45RA^+^ vs. CD45RO^+^ T cells; (**e**) MSC identification based on forward scatter (FSC) vs. side scatter (SSC); (**f**) CD73^+^, CD90^+^, and CD105 ^+^ vs. CD14^+^CD20^+^CD34^+^CD45^+^.

After 2 or 14 days in culture, the hBMNCs were washed with phosphate-buffered saline (рН = 7.2), and a cell suspension was mixed with a cocktail of mAbs against CD3, CD4, CD8, and CD25 (Abcam, Cambridge, UK); and CD45RO, CD45RA, CD71, and CD95 (e-Bioscience, Santa Clara, CA, USA). The CD45^+^CD3^+^ subpopulations were detected (Figure 2a–d).

After 14 days in culture, the hAMSCs were detached from the plastic wells with 0.05% trypsin (PanEco, Russia) in 0.53 mM EDTA (Sigma-Aldrich, St. Louis, MO, United States) and washed twice with phosphate-buffered saline. The surface markers on viable AMSCs were analyzed with a Human MSC Phenotyping Kit (cat. no. 130-095-198, Miltenyi Biotec, Bergisch-Gladbach, Germany), which detects CD14, CD20, CD34, CD45, CD73, CD90, and CD105 (Figure 2e,f).

After a 10-min incubation with the labeled mAbs, the cells were assayed using a MACS Quant flow cytometer (Miltenyi Biotec, Bergisch Gladbach, Germany) according to the manufacturer’s protocol. Flow cytometry (FC) results were analyzed using KALUZA Analysis Software (Beckman Coulter, Brea, CA, USA).

The in vitro viability of hBMNCs and hAMSCs was estimated with a MACS Quant flow cytometer (Miltenyi Biotec, Bergisch Gladbach, Germany). A total of 6 × 10^5^ cells was resuspended in binding buffer, and 5 µL of Annexin V:FITC (Abcam, Cambridge, UK) was added to 195 µL of the cell suspension. The cells were incubated for 10 min, washed, and resuspended in binding buffer.

An aliquot of 190 µL of the cell suspension was mixed with 10 µL of propidium iodide solution (Abcam, Cambridge, UK), and the resulting mixture was analyzed by FC. hAMSCs were preliminarily harvested with 0.05% trypsin (PanEco, Moscow, Russia) in 0.53 mM EDTA (Sigma-Aldrich, St. Louis, MO, USA) and washed twice with phosphate-buffered saline. The percentages of live and dead (apoptotic or necrotic) cells were measured according to the manufacturer’s protocol.

### 2.8. Cytokine Profile of Cultured Cells

Cell culture supernatants were collected on days 2 and 14 and centrifuged at 500× *g* for 10 min. FC was performed to measure the spontaneous and CaP coating-induced secretion of the following human cytokines and chemokines: Interleukin (IL)-1β, IL-1Ra, IL-2, IL-4, IL-5, IL-6, IL-7, IL-8, IL-9, IL-10, IL-12, IL-13, IL-15, IL-17, tumor necrosis factor alpha (TNFα), interferon gamma (IFNγ), eotaxin, granulocyte colony stimulating factor (G-CSF), granulocyte-macrophage colony-stimulating factor (GM-CSF), interferon gamma-induced protein 10 (IP-10; C-X-C motif chemokine 10 (CXCL10)), monocyte chemoattractant protein-1 (MCP-1; chemokine (C-C motif) ligand 2 (CCL2)), macrophage inflammatory protein 1 alpha (MIP-1α; CCL3), MIP-1β (CCL4), regulated upon activation, normal T cell expressed and secreted (RANTES; CCL5), basic fibroblast growth factor (bFGF), platelet-derived growth factor (PDGF-BB), and vascular endothelial growth factor (VEGF).

FC was conducted with mAbs according to the manufacturer’s instructions for the cytokine assay system (Bio-Plex Pro Human Cytokine 27-Plex Panel, Bio-Rad, Hercules, CA, USA) using an automated processing system (Bio-Plex Protein Assay System, Bio-Rad, Hercules, CA, USA). The concentration of each cytokine is presented in pg/mL.

### 2.9. Estimation of the In Vitro Osteogenic Differentiation of Cultured hAMSCs and hBMNCs

To establish the self-differentiation potential of cells in plastic wells and on a rough CaP surface, osteogenic supplements were not added to the culture medium. hAMSCs at a final concentration of 1.5 × 10^5^ live cells per 1.5 mL were cultured in 90% α-MEM (Sigma-Aldrich, St. Louis, MO, USA) supplemented with 10% fetal bovine serum (Sigma-Aldrich, St. Louis, MO, USA), 50 mg/L gentamicin (Invitrogen, Carlsbad, CA, USA), and 280 mg/L L-glutamine solution (Sigma-Aldrich, St. Louis, MO, USA) with or without the CaP-coated samples (cells were seeded on and around the samples) at 100% humidity with 5% CO_2_ at 37 °C for 21 days as described previously [20]; the medium was replaced with fresh medium every 3–4 days. hBMNCs at a final concentration of 1 × 10^6^ live cells per 1.5 mL of nutrient medium were incubated for 21 days as described above. The above concentrations of hAMSCs and hBMNCs were mixed, and the cells were cultivated as described above at a 6.7:1 ratio as reported previously [30,31].

The multipotent potential of hAMSCs was estimated by staining with alcian blue (Sigma-Aldrich, St. Louis, MO, USA) to visualize proteoglycan synthesis by chondrocytes, alizarin red S (Sigma-Aldrich, St. Louis, MO, USA) to identify mineralization of the extracellular matrix (ECM) by osteoblasts, and oil red (Sigma-Aldrich, St. Louis, MO, USA) to detect neutral triglycerides and lipids in adipocytes. hBMNCs and mixed cultures were stained with alizarin red S after 21 days of cultivation. All staining procedures were performed as recommended by the manufacturer. Adherent hBMNCs were also stained with fast blue PP salt (C_15_H_15_N_3_O_3_·BF_4_, m.w. 372.10; Lachema, Czech Republic) to detect alkaline phosphatase (ALP) activity after 3 days of culture as described previously by our group [32].

The results were assessed with a Zeiss Axio Observer A1 microscope (Carl Zeiss Microscopy, LLC, Oberkochen, Germany) using ZEN 2012 software (Carl Zeiss Microscopy, LLC, Oberkochen, Germany) on plastic surfaces and with a reflected light microscope (Olympus GX-71 metallographic device, Olympus Corporation, Tokyo, Japan) on CaP surfaces.

### 2.10. Statistical Analysis

Statistical analyses were conducted using the STATISTICA 13.3 software package for Windows (TIBCO Software Inc., Palo Alto, CA, USA). The mean (X) and standard deviation (SD) or median (Me) and 25% (Q1) and 75% (Q3) quartiles were calculated. The normality of the data distribution was defined by the Kolmogorov-Smirnov test. Because of the nonnormal data distribution, the nonparametric Mann–Whitney and Wilcoxon *T* test (*P*_T_) were used to evaluate significant differences between samples. Statistically significant differences were considered at *P* < 0.05. Relationships between the studied parameters were established via regression analyses. Significant relationships were indicated by coefficient (r) values with a significance level greater than 95%.

## 3. Results

### 3.1. Surface Topography Characterization

**Figure 3:**
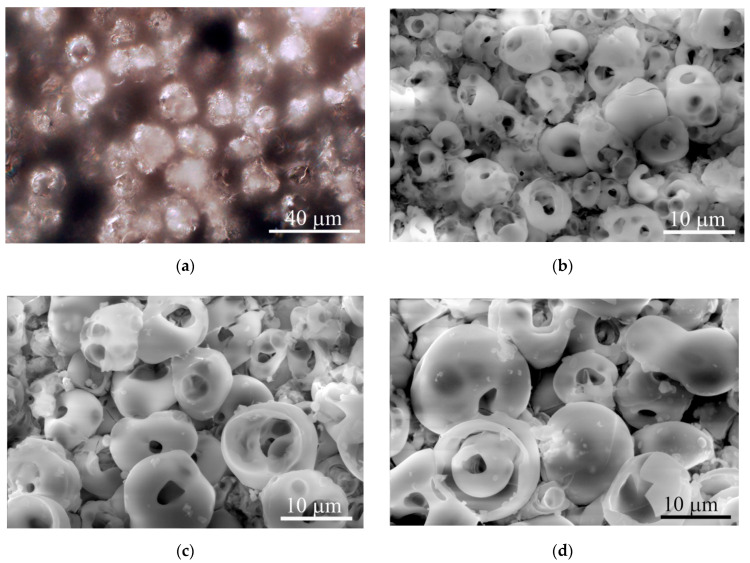
Optical microscopy (**a**) and SEM (**b**)–(**d**) images of typical topography of the microarc rough CaP coating on titanium substrates. (**a**) Reflected dark-field microscopy; (**b**) *R_a_* = 2.0−2.9 µm; (**c**) *R_a_* = 3.0−3.9 µm; (**d**) *R_a_* = 4.0−4.9 µm. Scale bars 10 and 40 µm. Magnification, × 1000 (**a**) and × 2500 (**b**–**d**).

The microreliefs of the CaP surface with *R_a_* = 2−5 µm had similar irregularities. The peaks of the CaP topography consisted of spherulites of up to 10–20 μm in diameter (Figure 3). The optical microscopy (Figure 3a) showed interconnected valleys as vast dark fields between ranges of bright spherulites; the areas are presented in Table 1. Single or open interconnected pores (1–10 μm in diameter) were observed by SEM in both spherulites (Figure 3b–d) and valleys independent of the roughness index.

Roughness indices calculated for the elevated (*R_a_*, *R_z_*) and horizontal (*S_m_*) profiles of the microarc CaP coating topography are presented in Table 1. A strong linear regression between *R_a_* and *R_z_* was identified (*r* = 0.94; *n* = 10; *p* < 0.00006). No regression was noted between *R_a_* and *S_m_* (*r* = −0.17; *n* = 10; *p* = 0.64). Additionally, we determined the areas of irregularly shaped surface valleys surrounding the spherulites of the microarc CaP coating (Table 1; Figure 3).

The regression analysis identified a strong relation between *R_a_* = 2−4 µm and S (*r* = 0.93; *n* = 16; *p* < 10^−6^; S (%) = 24.44 + 6.71x). Based on these data, *R_a_* seemed to be sufficient to characterize the roughness of microarc CaP-coated substrates for the biological experiments.

### 3.2. In Vivo Ectopic Vascularization of CaP-Coated Implants

**Figure 4:**
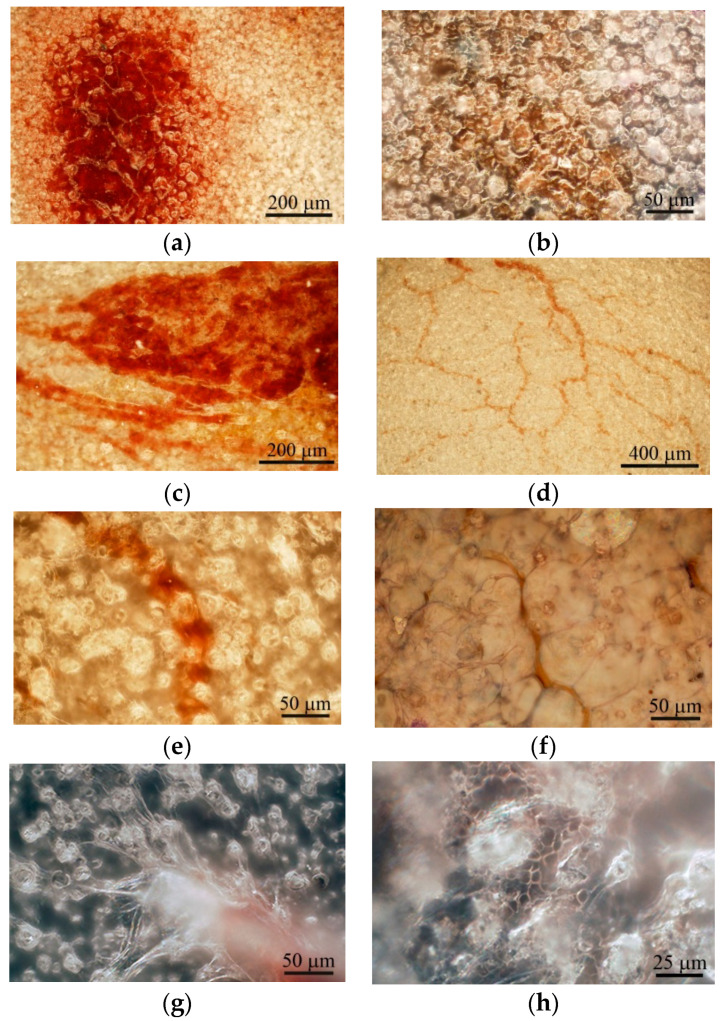
Surface of the microarc rough CaP coating on the titanium substrate after subcutaneous implantation in mice. (**a**) One; (**b**) two; (**c**) three; (**d**) four; and (**e**,**f**) five weeks after implantation. (**g**) Blood vessel stalks; (**h**) erythrocytes in capillaries between surface spherulites at 5 weeks after subcutaneous implantation. (**b**,**g**,**h**): Bright—and dark-field reflecting optical microscopy was used. Hematoxylin and eosin staining (**b**,**f**). Scale bars 25–400 µm.

There are technical difficulties with obtaining clear optical images of relief features with *Ra* > 3.5 µm, especially after removing implants from tissues. Therefore, CaP-coated titanium substrates with *R_a_* = 2−3.3 µm were used as the implants. Post-implantation disruption of the local vasculature within subcutaneous tissue resulted in a hematoma lasting 1–3 weeks (Figure 4a–c), which is critical for subsequent wound healing [33]. Long-lasting hematoma may be conditioned by the biomechanical forces (shear load and slight compression) exerted on the graft site and may be caused by lateral excursion of murine skin.

Microvessels were observed on the CaP coating near the hematoma at the end of the 3rd week after subcutaneous implantation (Figure 4c). The intensive vascular bed formed from pre-existing blood vessels (angiogenesis) [13] in surrounding soft tissue within 4 weeks (Figure 4d). Microvessel elongation (Figure 4e,f) occurred with the help of stalk and tip cells [13] that migrated on the CaP surface (Figure 4g).

In addition, some microvessels were observed between the spherulites in the surface valleys (Figure 4e,h), suggesting endothelial cell invasion into the CaP relief or possibly primary de novo capillary formation (vasculogenesis). Finally, blood vessels between adipose-like cells negative for hematoxylin and eosin (Figure 4f) were detected on the CaP coating relief at 5 weeks after subcutaneous implantation.

### 3.3. In Vitro Modeling of Microarc CaP Coating, hBMNCs and hAMSCs Connections

#### 3.3.1. Cytokine Secretion, Cell Viability and Cellular Immunophenotype

Close effective interactions between blood cells and resident cells, mainly hAMSCs, form the basis of successful vascularization, osteogenesis, and wound and fracture healing [1,33]. Therefore, cellular and molecular crosstalk among AMSCs, hBMNCs, and relief CaP material was modeled in vitro. There is a current trend towards modeling cell behavior under 3D culture conditions.

#### 3.3.2. hBMNC Culture

hBMNC secretory activity was established in 2-day and 14-day cultures (Tables S1 and S2 in Supplementary Materials). Increased concentrations of proinflammatory (IL-1β, IL-2, IL-6, IL-9, IL-15, IL-17) and anti-inflammatory (IL-1Ra, IL-12(p70)) ILs, granulocyte and/or monocyte/macrophage growth factors (G-CSF, GM-CSF), and chemokines (IL-8, MCP-1, MIP-1α, MIP-1β) were detected after 2 days of hBMNC cultivation in the presence of CaP-coated samples (3D culture) compared with cell culture on plastic (control 2D culture). Conversely, the secretion of angiogenic molecules (VEGF, PDGF-BB, basic FGF (bFGF)) did not differ significantly between 2D and 3D culture (Table S1).

The nutrient medium was replaced every 3–4 days under conditions of prolonged cell culture. Therefore, the concentrations of some biomolecules (IL-2, IL-5, IL-7, IL-15, IL-17, G-CSF, VEGF) were not detected after 14 days of cultivation due to depletion of hBMNC secretion (Table S2).

CaP-coated samples served as cell irritants and sharply increased (3–100 times; P_T2_ < 0.05) the levels of most of the tested humoral factors, excluding IL-5, IL-7, and IL-1Ra. IL-15 was produced de novo by 3D-stimulated hBMNCs (Table S2).

As shown in Table S3 in Supplementary Materials, CD45^+^CD3^+^ hBMNCs in 2-day 2D control culture expressed a wide range of membrane markers, predominantly CD45RA, an indicator of naïve (no antigen activation) CD4^+^ T helper/inducers. The membrane activation and costimulatory molecules CD25, CD71, and CD95 were present on 9, 2.5, and 15% of CD45^+^CD3^+^ cells, respectively. Thirty-five percent of CD45^+^CD3^+^ cells were positive for the CD45RO isoform of this transmembrane antigen of activated T lymphocytes and/or memory T cells. Surprisingly, 62% of hBMNCs were CD73^+^ after 3 days of culture. A very small percentage of cells (less than 1%) were positive for other stromal markers (CD90 and CD105) (Table S4 in Supplementary Materials).

hBMNCs from other healthy volunteer were cultured for 14 days. These cells showed markedly increased levels of CD25, CD71, and CD95, unlike both freshly isolated hBMNCs (see 2.3) and hBMNCs cultured for 2 days under control 2D conditions (Tables S3–S5 in Supplementary Materials).

Interestingly, short and long contact of hBMNCs with CaP-coated samples did not significantly change the spectrum of tested leukocyte antigens on CD45^+^CD3^+^ cells (Tables S3–S5). At the same time, a relative increase in the number of CD73^+^, CD90^+^, and CD105^+^ cells or CD45^+^ hBMNC subsets with hematopoietic but not monocytic CD14 antigens was detected at as late as 3 days of cultivation (Table S4).

The viability of the nonadherent fraction of hBMNCs diminished progressively because of a 2.5- to 4-fold increase in cell necrosis from contact with CaP-coated samples (Tables S3 and S5). In turn, the lower percentage of live hBMNCs at 14 days compared with 2 days in control 2D culture stemmed from a 7-fold increase in apoptosis (Tables S3 and S5). These results suggest the hyperactivation-dependent death of nonadherent T cells in contact with CaP-coated samples for 2–14 days.

#### 3.3.3. hAMSC Culture

The secretory activity after 14 days of cultivation (2D control 1) in a monolayer was obviously (10–1000) higher for hAMSCs than for hBMNCs regarding the studied cytokines and chemokines, excluding PDGF-BB (Table S2). For example, the VEGF concentration was increased up to 1814 pg/mL in hAMSC cultures versus 0 pg/mL in control 2D cultures of hBMNCs.

In contrast with 3D culture of hBMNCs, the concentrations of biomolecules (except IL-7) were decreased by 1.5- to 100-fold in hAMSC cultures containing CaP-coated samples compared with 2D stromal cell cultures. In addition, the concentrations of most cytokines and chemokines (IL-1β, IL-1Ra, IL-2, IL-9, IL-17, TNFα, bFGF, PDGF-BB, GM-CSF, IL-8, IP-10, MIP-1α, MIP-1β, RANTES) were lower in hAMSC 3D culture than in hBMNC 3D culture. A few proinflammatory (IL-6, IL-7), angiogenic (VEGF), and antiangiogenic (IL-12p70) biomolecules were at higher levels in hAMSC cultures containing the CaP-coated samples than in hBMNC 3D cultures (Table S2).

The immunophenotype of hAMSCs in control 2D culture (more than 91% CD73^+^, CD90^+^, or CD105^+^ cells) corresponded to the MSC profile. Fourteen days of contact between hAMSCs and the rough CaP coating decreased (by 4–5%) the percentage of cells expressing stromal markers, mainly CD73 and CD105 (Table S6). Hematopoietic antigen presentation was unchanged. Overall, the rough CaP-coated titanium substrates significantly diminished hAMSC apoptosis and necrosis, leading to an increase in cell viability (Table S6).

#### 3.3.4. hAMSC and hBMNC Allogenic Coculture

The concentrations of all tested biomolecules were higher in 14-day mixed hAMSC+hBMNC allogeneic cultures (2D control 2) than in hBMNC monocultures according to the Wilcoxon *T*-test (P_T_ < 0.05) (Table S2). Moreover, significantly elevated levels of most ILs (excluding IL-4, IL-7, and IL-15), cytokines (excluding IFNγ), hematopoietic growth factors, angiogenic molecules (except PDGF-BB) and chemokines (eotaxin only) were detected in 2D coculture compared with hAMSCs alone (2D control 1). Conversely, the MIP-1α concentration decreased (Table S2).

CaP-coated titanium specimens had predominantly costimulatory effects on the secretory activity of hBMNCs and hAMSCs in mixed 3D culture (*P*_T_ < 0.05 compared with 3D monocultures). The increased concentration of the anti-inflammatory molecule IL-1Ra and the 3-fold decreased concentration of the proinflammatory molecule IL-6 in mixed 3D culture compared to 2D coculture suggest inflammatory signal switching in favor of regeneration pathways in the presence of the CaP coating (Table S2).

The immunophenotype of CD45^+^CD3^+^ leukocytes was changed in mixed hAMSC+hBMNC allogeneic 2D culture (Table S5). The fractions of CD4^+^ T helper/inducers and CD45RO^+^ activated T lymphocytes and/or T memory cells increased by 2-fold and 12%, respectively, in contrast to the significantly decreased percentages of CD25^+^ and CD71^+^ hBMNCs (Table S5). Furthermore, decreased percentages of hAMSCs positive for CD73 (from 96.55 to 93.33%) and CD105 (from 92.94 to 90.83%) were observed in mixed culture (2D control 1) (Table S6).

The introduction of CaP-coated specimens to hAMSC+hBMNC cultures did not significantly influence the antigen spectrum of hBMNCs in the mixed cell population. In general, the changes in molecular expression on hBMNCs were the same in mixed 2D and 3D cultures as in the corresponding leukocyte monocultures. Increased percentages of CD4^+^, CD95^+^, and CD45RO^+^ or CD45RA^+^ leukocytes and decreased percentages of CD71^+^ and CD25^+^ leukocytes were detected. In addition, the relative number of CD95^+^ cells in mixed 3D culture was significantly different from the corresponding cell number in hBMNC 3D culture (Table S5).

CD73 presentation on hAMSCs was reduced by 5% in mixed 3D culture compared with 2D coculture (Table S6). Both 2D and 3D cocultures strongly increased hBMNC viability to up to 97% (Table S5), and CaP-coated substrates increased hAMSC viability under both monoculture and mixed culture conditions (Table S6). In vitro, hAMSCs promoted the long-term viability of allogeneic hBMNCs, especially those positive for CD4. The rough CaP coating sustained the vitality of hAMSCs but not hBMNCs.

#### 3.3.5. In Vitro Osteogenic Differentiation

**Figure 5:**
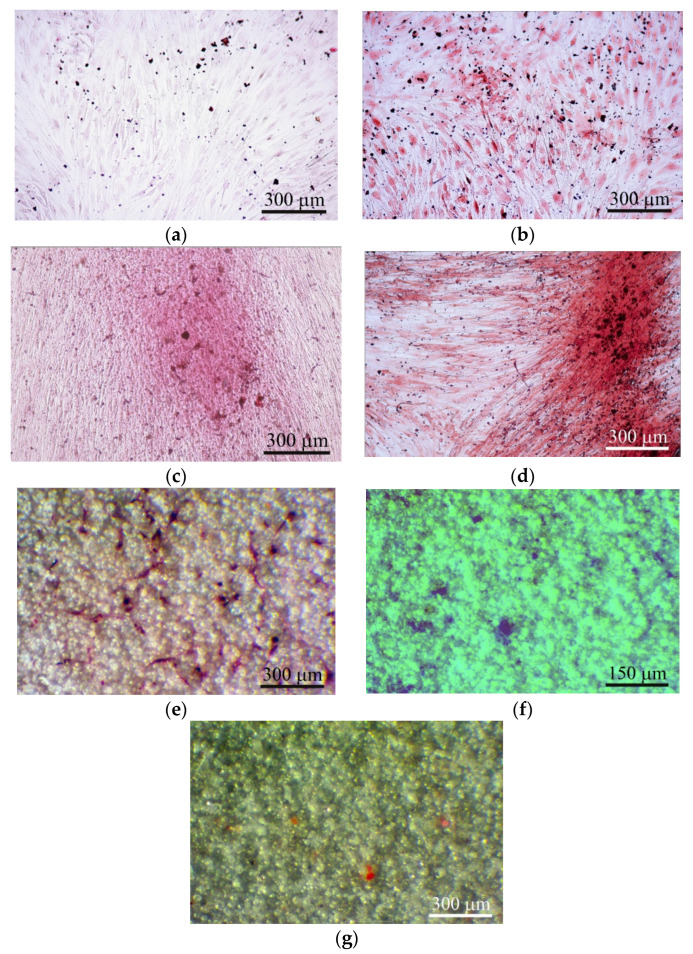
hAMSC culture after 21 days of culture on plastic surface (**a**)–(**d**) and on the rough CaP coating (**e**)–(**g**) in a standard nutrient medium**.** (**a**) 2D culture of hAMSCs; (**b**) culture of hAMSCs around CaP-coated specimens; (**c**) mixed 2D culture of hAMSCs+hBMNCs; (**d**) mixed 3D culture hAMSCs+hBMNCs around CaP-coated specimen; (**e**) Sites of the cell and extracellular matrix (ECM) mineralization. Alizarin red staining (**a**)–(**e**); (**f**) alcian-blue-stained sites of glycoproteins; (**g**) oil-red-stained sites of neutral triglycerides and lipids. Scale bars 150 and 300 μm.

Adherent fibroblast-like CD73^+^CD90^+^CD105^+^ hAMSCs cultivated on plastic wells in standard nutrient medium for 21 days stained poorly with alizarin red S (Figure 5a). The differentiation of hAMSCs into osteoblasts on plastic around CaP-coated specimens was enhanced, but only diffuse alizarin red staining was observed (Figure 5b).

Alizarin red staining of individual cells did not allow us to calculate the area of ECM mineralization in hAMSC monocultures (Table 2). Staining appeared when hBMNCs were added to hAMSCs (Table 2; Figure 5c), and a 10-fold increase in the total area of ECM mineralization around the CaP-coated samples was detected in the mixed culture (Table 2; Figure 5d).

The marked increase in the number of sites of ECM mineralization on plastic in 3D mixed culture compared with control 2D mixed culture indicates an increase in hAMSC differentiation into osteoblasts in response to indirect contact with the CaP coating.

Interestingly, satisfactory osteogenic and chondrogenic differentiation (Figure 5e,f) but poor adipogenic (Figure 5g) differentiation of hAMSCs on the rough CaP surface were detected by differential cell staining. The microarc CaP coating and its biodegradable products can switch hAMSC fate.

**Figure 6:**
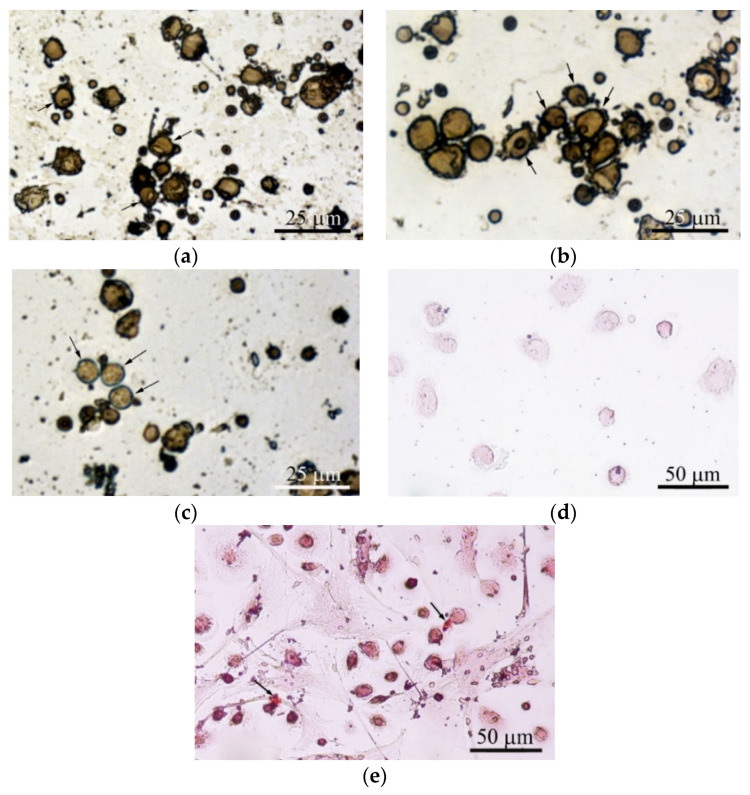
hBMNC monocultures after 3 days (**a**)–(**c**) and 21 days (**d**), (**e**) in a standard nutrient medium. (**a**) 2D culture of hBMNCs on plastic surface; (**b**), (**c**) 3D culture of hBMNCs around CaP-coated specimen. Alkaline phosphatase (ALP) staining with fast blue PP salt. Some cells with nucleoli (**a**), (**b**) and single adherent ALP-positive cells with blue stained cytoplasm (**c**) are marked by black arrows; (**d**) adherent round cells; (**e**) adherent round and fibroblast-like cells. Small sites of mineralization stained with alizarin red are shown by black arrows. Scale bars 25 and 50 μm.

Adherent hBMNCs in 3-day culture on plastic (2D culture) have been shown to adopt two phenotypes, large (diameter greater than 20 µm) cells with membrane pseudopodia and small (10 µm or smaller) round cells. Some cells with nucleoli were blast cells capable of proliferation (Figure 6a). The presence of a microarc CaP coating increased the number of irregularly shaped large hBMNCs with nucleoli (Figure 6b). In addition, single ALP-positive cells with blue-stained cytoplasm were noted in 3D culture (Figure 6c).

The macrophage-like shape of hBMNCs was accompanied by the increased expression of stromal antigens (CD90, CD105, and especially CD73) but not CD14 (Table S4). hBMNCs secreted a notable concentration of angiogenic molecules (PDGF-BB) after 14 days of cultivation (Table S2). Round hBMNCs in 21-day 2D cultures were negative for alizarin red staining (Figure 6d).

However, adherent cells (0–1.6% per well) had a fibroblast-like shape. Surprisingly, single small sites of alizarin red staining (ECM mineralization) were observed at places of contact between round and fibroblastoid cells (Table 2; Figure 6e). Therefore, the data suggest that CaP-coated samples caused the accumulating biomass of CD73^+^ endothelial cells (angioblasts) and/or MSCs/osteoblasts in adherent hBMNC culture (Figure 6b,c).

Most likely, the blood circulating fraction of stromal cells among hBMNCs is capable of osteogenic differentiation during 21 days of in vitro culture. hBMNC populations have been proposed as a source of enhanced ECM mineralization in mixed culture with hAMSCs. Nevertheless, their contribution was unappreciable versus hAMSCs (Table 2), and hBMNC participation is initially conditioned by a spectrum of secreted molecules (Tables S1 and S2).

#### 3.3.6. Cell-IQ Visualization of Cell Behavior

Cell-IQ continuous real-time automated monitoring did not show significant differences in the average velocity of cell division (AVCD) between the experimental groups (Table 3). No changes in AVCD were determined at any point in the 6 visualization fields (see Figure 1) (data not shown).

Therefore, hBMNCs did not affect hAMSC division or viability (Table 3), and cell biomass alterations in the experimental groups (Table 4) were exclusively related to stromal cell migration. Different numbers of hAMSCs were counted in various visualization fields at the end of the observation period (Table 4) because of unequal initial cell distribution and irregular cell motility: Cells migrated in or out of visualization fields. The findings for all six visualization fields are presented in Table 4.

The data showed limited hAMSC movement near the initial cell drop (1–2, 4–5; Figure 1) caused by CaP-coated samples and/or hBMNC addition. Both the CaP coating and the presence of hBMNCs had synergistic negative effects on hAMSC motility (Table 4). This effect of the microarc CaP coating stemming from its dissolution was reported previously [27].

**Figure 7:**
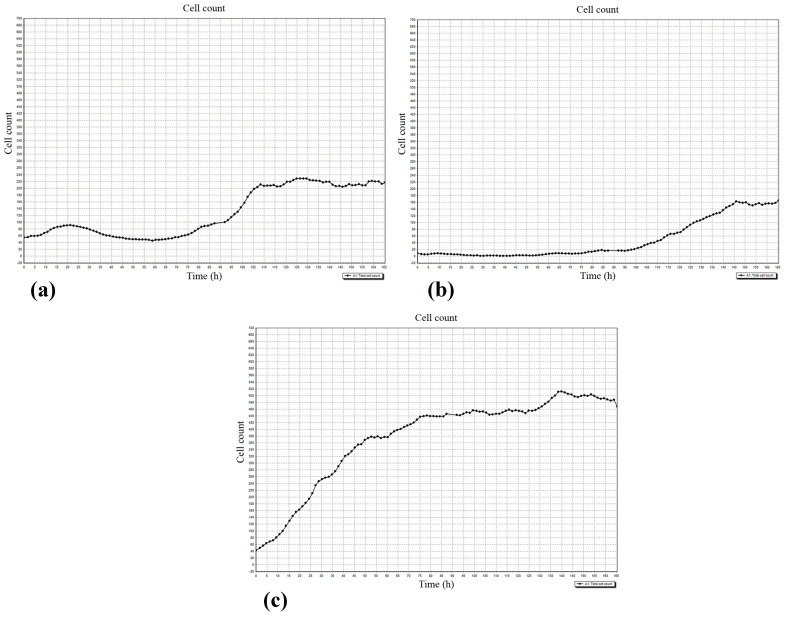
The dynamics of cell accumulation in the 5th visualization field (see Figure 1) of the Cell-IQ system. (**a**) hBMNCs; (**b**) hAMSCs in mixed cell culture; (**c**) hAMSCs in monoculture. *X*-axis: Observation time (h); *Y*-axis: Cell count.

The regulatory effect of hBMNCs on hAMSC mobility was confirmed by the concordant graphs of leukocyte (Figure 7a) and stromal cell (Figure 7b) count, and the number of hAMSCs was below that in stromal cell monoculture (Figure 7c). The data indicated that hBMNC chemokines (Tables S1 and S2) attract hAMSCs, and this hypothesis was verified by the RTCA experiments (see below).

Note: *n* = number of wells in each plate for each group; *n_1_* = number of dividable cells.

#### 3.3.7. Cell Invasion and Recruitment

**Figure 8:**
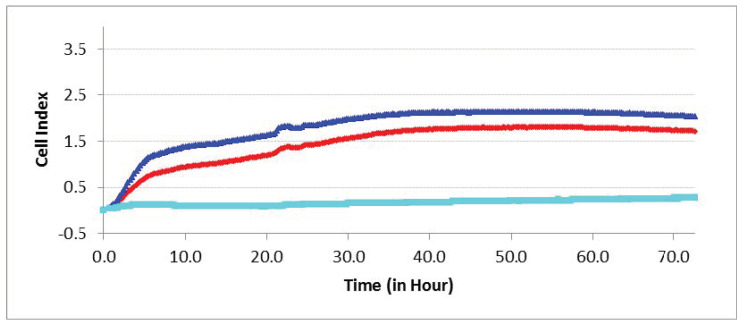
Experimental curves of impedance in the real-time cell analyzer (RTCA) system mirror the migration index (MI) of different cells passing through the microporous membrane. hAMSC invasion towards hBMNCs (top blue line), and vice versa (bottom cyan line); control hAMSC motility towards cell-free nutrient medium (middle red line).

Enhanced (P_T_ < 0.05) hAMSC invasion towards hBMNCs compared with cell-free medium was shown for 3 days in the RTCA experiments (Figure 8). Conversely, hBMNC motility conditioned by indirect (humoral) contact with hAMSCs remained at basal levels.

Therefore, chemokines secreted by hBMNCs (Tables S1 and S2) promote hAMSC recruitment into sites of inflammation and/or regeneration.

## 4. Discussion

Artificial surface topography is known to have an important effect on cell behavior [34], and the roughness of CaP coatings prepared on titanium substrates by MAO method controls MSC fate [20]. Biocompatible microarc CaP coatings [35] with a fixed *R_a_* = 2–4 µm promote MSC osteogenic differentiation both in vitro [20] and in vivo [36]; therefore, these conditions were adopted in our experiments. The microstructure, phase, and elemental composition, as well as physicochemical and mechanical properties were described for microarc CaP coatings in [37], in relation to the applied voltage in the range from 200 to 350 V. According to these results, the microarc CaP coatings on titanium substrate had mainly an amorphous microstructure with the CaHPO_4_ phase for all applied voltages. The increase in the MAO voltage led to a coating structure transformation from X-ray amorphous to the amorphous-crystalline state. Thus, chemical features of microarc CaP coatings excluding, to some degree, the Ca/P atomic ratio were not depended on the increasing magnitudes of applied voltage [37].

On the other hand, surface and bulk features (*R_a_*, mass and thickness) are very processible and connected closely with the applied voltages and biological reactions [6,20,37,38,39]. Because some variations of calcium, phosphorus, oxygen, and titanium contents and phase compounds accompanied with surface and bulk indices [37], microarc CaP-coated samples with a fixed range of *R_a_* = 2–5 µm were used in the different series of experiment ( Table 1, Table 2, Table 3 and Table 4, Tables S1–S6).

There are numerous reviews regarding the biological impact of *R_a_* index of surface roughness in vitro [40] or in vivo [41]. However, relative investigations are obviously insufficient. Zigterman et al., (2019) believe that the in vitro research methods differed too much from the in vivo research methods for reliable comparison of the results [41]. Therefore, our investigation is of apparent interest for materials scientists and biologists in the field of microarc CaP coatings and their biomedical applications.

### 4.1. Relationships between CaP Surface Roughness Indices

There is a strong direct dependence of *R_a_* on voltage from 150 to 400 V, a key technological parameter of the MAO technique [38]. A close linear regression between *R_a_* and *R_z_* of the microarc CaP coating was estimated (see Section 3.1). In turn, *R_a_* is correlated with indices of the osteogenic differentiation and maturation of MSCs on microarc CaP coatings in vitro [39] and in vivo [6]. No regression between *R_a_* and *S_m_* was found in this study (see Section 3.1). Thus, our previous conclusion [20] that only the roughness index *R_a_* (and not *S_m_*) can be controlled technologically during the CaP coating of titanium via MAO was confirmed. Certain authors consider *S_m_* (the linear distance between surface features (surface peak and valley widths)) the more convenient parameter of surface topography to characterize the relationship between surface roughness and in vitro hMSC adhesion on metallic substrates [40]. The situation for dielectric CaP materials is different.

Furthermore, we calculated the area (S) of irregularly shaped surface valleys surrounding the spherulites of the micro-arc CaP coating (Table 1; Figure 3) because they are preferred by osteogenic cells in vitro [20,32]; these valleys were occupied by blood microvessels in the ectopic experiment in vivo (see Section 3.2; Figure 4e,h). A close relation between *R_a_* = 2−4 µm and S was detected (see Section 3.1). Adherent hMSCs range widely in size by origin, for example, 20–30 µm from prenatal lung [32] and more than 100 µm from fat (Figure 5b) and blood (Figure 6e), and respond to surface features relative to cell size and seed surface microterritories with highly divergent properties. In this regard, the relative (percentile) dimensions of surface valleys in implants should have biological significance.

According to our developing concept of “niche-relief” for MSCs [42], these areas of surface valleys can be considered important technological, physical, and biological features of anisotropic microarc rough CaP coatings on titanium substrates.

### 4.2. In Vivo Contradictions of Subcutaneous Ectopic Implantation

The formation of new blood vessels from pre-existing blood vessels (angiogenesis) is critical for the biocompatibility/biodegradation of implanted biomaterials and their possible ossification [13]. Despite poor subcutaneous vascularization and blood flow compared with muscle and kidney capsules [1], inorganic biomaterials, mainly CaPs, can induce direct ectopic bone formation (osteoinduction) without the addition of osteogenic cells or bone growth factors while implanted under the skin [1,9,10]. In contrast, there are reports of CaP scaffold failure after subcutaneous implantation [10]. A few investigations described subcutaneous osteoinduction induced by CaP implantation in mice with a low incidence (3/16) of new bone formation [43]. The probability of material-induced bone formation has been concluded to vary with animal species and to be related to the physical-chemical features of the implant material [10,43].

Therefore, our study of the in vivo vascularization of rough CaP-coated samples implanted subcutaneously in mice was in line with current trends in this field. Post-implantation disruption of the local vasculature within mouse subcutaneous tissue resulted in a hematoma lasting 1–3 weeks (Figure 4a–c) that enabled wound (fracture) healing, revascularization of the injured region [33], and new bone formation [44] when the hematoma was explanted to an ectopic site [45].

In addition to its osteogenic potential, a hematoma possesses angiogenic features [46]. The fibrin network provides a microenvironment for various cellular functions, such as the migration, proliferation, differentiation, and maturation of blood, endothelial, and stromal cells, as well as their secretion of biomolecules. Cells and cytokines initiate the cascade of events essential for revascularization [44].

Indeed, microvessels around the hematoma were found on the rough CaP coating at 3 weeks after subcutaneous implantation in mice (Figure 4c). The intensive vascular bed formed from pre-existing blood vessels (angiogenesis) [13] in surrounding soft tissues after 4 weeks of observation (Figure 4d).

Angiogenic growth factors activate endothelial cell receptors in existing blood vessels. Upon stimulation by proangiogenic gradients, activated cells release proteases that allow them to escape from the blood vessel walls and to migrate, proliferate and form sprouts connecting neighboring vessels. The tandem migration of endothelial cells forms cellular loops that become vessel lumens. These sprouting networks develop stalk cells and a leading tip cell that guides the migration of the developing vessel into surrounding tissue towards chemotactic gradients. The stalk cells, on the other hand, elongate the vessel at a rate of several millimeters per day, as reviewed previously [13,47]. In this context, our in vivo experimental results indicate microvessel elongation with the help of stalk and tip cell migration on the rough CaP surface (Figure 4g).

There are two time points for revascularization: an early time point on the order of days (at the end of the inflammatory phase) and a later time point at approximately 3 weeks (the beginning of bone formation) [48]. Late angiogenesis is likely a critical reason for the delayed (after 13 weeks) and inconsistent bone formation (0–3/16) by subcutaneous EO in mice implanted with CaP materials without osteogenic cells or bone growth factors [43].

Unlike angiogenesis, de novo vasculogenesis involves endothelial precursor cell (angioblast) migration to form new primary capillaries in response to gradients of angiogenic growth factors and signaling molecules [49,50]. VEGF, bFGF, and PDGF represent the most prominent molecules that stimulate angiogenesis and vasculogenesis [51]. In our study, some microvessels grew between the spherulites in the surface valleys (Figure 4e,h), which suggests endothelial cell invasion into the rough CaP relief or possibly de novo primary capillary formation, which was termed vasculogenesis by Risau [52].

Numerous groups have found that macroporosity (pore size larger than 30–40 µm) greatly influences the vascularization of ceramic scaffolds [53] through the secretion of proangiogenic factors by cells in contact with CaP materials [53]. Similarly, the microarc rough CaP coating on titanium substrates with comparable topographical elements and *R_a_* = 2−4 µm [20] has an angiogenic effect (Table S1; Figure 4).

Finally, vascularized tissue with adipose-like cells negative for hematoxylin and eosin covered the rough CaP coating at 5 weeks after subcutaneous implantation (Figure 4f). Therefore, a question regarding interactions between cells of fat origin and the CaP coating has arisen despite the poor (1 of 9 cases) EO features of hAMSCs not treated with recombinant BMP2 and subcutaneously implanted in immunodeficient mice on porous CaP ceramic particles [54]. At 8 weeks, weak vascularization and EO induced by mouse AMSCs immobilized on bone substitute material Bio-Oss were reported [55].

At the same time, adipose tissue itself as a scaffold for AMSC expansion resulted in ectopic bone tissue formation through endochondral ossification at 8 weeks after in vivo implantation [56].

### 4.3. A Question Regarding in Vitro Models of hBMNC and AMSC Participation in Subcutaneous Angiogenesis and EO

BM-MSCs and AMSCs are commonly used sources in skeletal tissue engineering in vitro and in vivo [4]. AMSCs from subcutaneous fat in the perivascular compartment promote local angiogenesis [17] and tissue formation as precursors of pericytes and other cells (adipocytes, osteoblasts, chondrocytes, endothelial cells, myocytes, etc.) [18] and secrete multiple cytokines and chemokines [17]. At the same time, little is known about the tissue origin (e.g., fat, skin, blood) of cells that can initiate angiogenesis and bone formation in subcutaneously implanted materials [1,9,10,11] without the addition of osteogenic cells or bone growth factors.

Skin injury results in vasculogenesis [13] and the homing of blood progenitor cells [14] and leukocytes from circulation to defects. On that basis, circulating stem/progenitor cells and pericytes are possible candidates for osteoblasts under conditions of EO [16].

Nevertheless, poor subcutaneous vascularization and blood flow in implants compared with muscle and kidney capsules [1] are obvious limitations for the massive participation of circulating MSCs and pericytes in subcutaneous osteoinduction on the implant surface and/or bulk before blood flow is restored.

Thus, in vitro modeling of the cellular and molecular crosstalk among the rough CaP material, blood leukocytes and AMSCs was of great interest to determine the secretion of angiogenic molecules by AMSCs and their osteogenic potential under both unstimulated and irritant-stimulated conditions.

There are some difficulties in murine MSC isolation, purification and cultivation [57]. Because of these challenges and the clinical need for stem cells for wound and bone healing, hAMSCs were used in this study.

Immune cells are vital modulators of inflammation, bone formation and angiogenesis [33]. After the development of a hematoma (Figure 4a–c), the migration of blood cells towards the implant surface is one of the first steps in subcutaneous healing. We have studied the in vitro cellular and molecular responses of hBMNCs on rough CaP coatings after a few days and after 14–21 days.

### 4.4. In Vitro Modeling of Cellular and Molecular Crosstalk between the Rough CaP Coating and hBMNCs

hBMNCs that migrate into the injured tissues are considered a source of mononuclear leukocytes, including lymphocytes and monocytes [33], and circulating stem/progenitor cells [16]. Leukocytes secrete various pro—and anti-inflammatory cytokines, chemokines, and growth factors to recruit additional inflammatory cells, promote neovascularization, direct MSC migration and differentiation, and mediate tissue remodeling [33] predominantly by promoting chronic inflammation within 2 weeks [10,43].

Indeed, hBMNCs in vitro generated a wide spectrum of inflammatory ILs, granulocyte and/or monocyte/macrophage growth factors, angiogenic molecules and chemokines after 2 days of culture (Table S1). Biomolecule secretion seemed to be depleted significantly at 14 days, especially for IL-2, IL-5, IL-7, IL-15, IL-17, G-CSF, and VEGF, which were not detected at this timepoint (Table S2). These findings assume long-term hBMNC activity with a changing array of secreted molecules, particularly more decreased proinflammatory cytokine and chemokine production versus anti-inflammatory IL-1Ra release, despite the potential diversity in the functional activity of hBMNCs collected from different healthy volunteers. Our results correspond to the current view about immune cells as regulators of inflammation/tissue repair switching [33], culminating in the emerging term “osteoimmunology” [58,59].

The interactions of lymphocytes and monocytes/macrophages with MSCs have been actively investigated [33,58,60,61]. Moreover, previous studies showed that CaP biomaterials can mediate the pronounced secretion of chemokines and cytokines by immune cells [29]. Complex experiments suggest measuring the secretion of proangiogenic factors by cells cultured in the presence of CaP materials [53].

Our in vitro experiment clearly showed that culturing hBMNCs with CaP-coated titanium samples markedly increased (3–100 times) the levels of secreted humoral factors (except IL-5, IL-7, and IL-1Ra) over a long time frame (14 days). The hBMNCs cultured for 14 days in 3D conditions showed a significant growth (relative to the 2-day culture) in the release of angiogenic molecules (VEGF, bFGF, PDGF-BB) compared with those cultured on a plastic surface (Tables S1 and S2). The rough CaP coating did not affect the expression of the tested lymphocytic determinants on CD45^+^CD3^+^ T cells (Tables S3 and S5) or the monocytic CD14 antigen on CD45^+^ leukocytes (Table S4).

It should be emphasized that the 7-fold increase in the apoptosis of nonadherent hBMNCs from 2–14 days of culture was enhanced by the rough CaP coating, which increased the signs of necrotic cell death (Tables S3 and S5). Together with the abovementioned data on cytokine secretion and membrane marker presentation, the data suggest the hyperactivation-dependent death of nonadherent T cells [62] in contact for 14 days with CaP-coated samples.

Surprisingly, an increase in the extremely large percentage of CD73^+^ cells (from 62 to 67%, Table S4) was observed upon contact with the rough CaP coating in the 3-day hBMNC culture. CD73 has been reported to be a surface antigen of cytotoxic, helper and regulatory T cells [63]. Quast et al., (2017) stated that CD73 expression on T cells is an important anti-inflammatory signal associated with the reduced production of proinflammatory molecules (IL-3, IL-6, IL-13, IL-17, MIP-1α, MIP-1β) [64]. Apparently, the rough CaP coating may serve as a physicochemical switch for T cell activity from pro- to anti-inflammatory, thus regulating angiogenesis and tissue regeneration.

In addition, CD73 is present on endothelial progenitors [65]. This study identified an increasing number of features of stromal cells, not CD14^+^ macrophages, such as small fractions of CD90^+^ and CD105^+^ hBMNCs, morphological signs of dividing cells (nucleoli), ALP-positive staining (3-day culture), and osteoblast-like properties (21-day culture, alizarin red staining) with a fibroblast-like shape in the angiogenic microenvironment (notable PDGF-BB levels) (Tables S1, S2 and S4; Figure 6). Therefore, the number of CD73^+^ endothelial cells (angioblasts) and/or MSCs/osteoblasts among hBMNCs increased by CaP-coated samples (Figure 6b,c) and accumulated over 14–21 days in the in vitro model of subcutaneous injury was proposed. Most likely, the fraction of stromal cells among circulating hBMNCs (Figure 6e) can serve as a source of circulating MSCs/progenitor cells as described previously [16]. Nevertheless, their paucity renders their contribution inappreciable compared with hAMSCs, and hBMNC participation revolves primarily on the secretion of a spectrum of inflammatory mediators.

### 4.5. In vitro Modeling of Cellular and Molecular Crosstalk between the Rough CaP Coating and hAMSCs

The in vitro and in vivo advantages of AMSCs over BM-MSCs mainly include enhanced angiogenesis that may be negated by their lack of EO [66,67] and the prerequisite for osteogenic differentiation by osteogenic priming [68] or gene manipulation [69] before transplantation and implantation on different scaffolds.

A limited number of publications have shown delayed (after 8 weeks) subcutaneous EO by unprimed hAMSCs on CaP (beta-tricalcium phosphate) material [70]. The question of whether AMSCs are capable of osteoinduction remains unresolved, and additional investigations and models are necessary.

Our in vitro experiment showed higher secretory activity of hAMSCs compared with hBMNCs that strongly decreased (Table S2) and increased viability of 14-day stromal stem cells (Table S6) cultured in the presence of the microarc rough CaP coating. The decreased secretion of angiogenic cytokines by hAMSCs in contact with rough CaP-coated substrates may be one reason for the late vascular bed formation on the implant surface at 4 weeks after transplantation (Figure 4d). The experimental samples promoted enhanced but diffuse in vitro staining of hAMSCs with alizarin red S around the CaP surface (Table 2; Figure 5a,b). Furthermore, the rough CaP coating did not influence hAMSC division (Table 3) but decreased the number of CD73^+^CD90^+^CD105^+^ cells (Table S6), restrained their motility (Table 4) and increased alizarin red staining (Figure 5e).

Such changes in hAMSCs favored their osteogenic differentiation after 21 days of contact with CaP-coated substrates. Overall, osteoinduction significantly inhibits the release of cytokines, chemokines, and growth factors by AMSCs, as confirmed by our results [71]. The absence of nodules of ECM mineralization (calcium deposition) around CaP-coated substrates in hAMSC monocultures (Figure 5a,b), in contrast to our previous results [20,27], emphasizes the variability in osteoinduction initiated in vitro and in vivo by various sources of hAMSCs [66]. Together with the delayed blood vessel formation (Figure 4), the data suggest that this model may induce poor subcutaneous EO that is not comparable with the results of other techniques (intramuscular and kidney capsule transplantation) [1].

On the other hand, the microarc rough CaP coating was able to directly switch the fate of individual hAMSCs from adipogenic to chondrogenic and osteogenic differentiation (Figure 5e,f); such fates are strongly enhanced in vitro by humoral osteogenic supplements, such as β-glycerophosphate, dexamethasone, and ascorbic acid [20].

In this context, vascularized fat tissue covering the CaP coating at approximately 5 weeks after implantation (Figure 4f) retained good osteogenic potential as described previously [56] through endochondral ossification at 8 weeks after in vivo implantation.

### 4.6. In Vitro Modeling of Cellular and Molecular Crosstalk among the Rough CaP Coating, hBMNCs and hAMSCs

The immunomodulatory properties of AMSCs are not completely understood. MSCs attenuate immune responses through their prevalent immunosuppressive capabilities stemming from the secretion of various biologically active molecules with immunomodulatory effects [30]. They can suppress [72] or not reduce [30] the proliferation of peripheral BMNCs in mixed allogeneic or xenogeneic culture, as well as stimulate the activation and proliferation of resting T cells and generate regulatory T cells [73].

The present study showed that 14 days after seeding, mixed hAMSC + hBMNC allogeneic cultures were characterized by a significant increase in the secretion of most tested cytokines, growth factors, angiogenic molecules, and osteomodulatory molecules, with a lesser effect on chemokines (eotaxin only), compared with hBMNC or hAMSC monocultures (Table S2 and Table 5).

Generally, CaP-coated titanium specimens in mixed 3D culture maintained the costimulatory effect of hBMNCs on the secretory activity of hAMSCs (P_T_ < 0.05), with effects in some cases (IL-1Ra, IL-17, bFGF, PDGF-BB, GM-CSF, MIP-1α) compared with mixed 2D culture (Table S2). The increased concentration of the anti-inflammatory molecule IL-1Ra and the 3-fold decrease in the proinflammatory molecule IL-6 in 3D mixed culture compared to 2D coculture indicate signal switching from acute inflammation to regeneration pathways in the presence of the rough CaP coating (Table S2). Moreover, there was enhanced production of ILs (P_T_ < 0.05), including mediators with angiogenic properties, and other angiogenic factors (VEGF, TNFα, G-CSF) (see references in Table 5) in 3D cocultures compared with 3D hBMNC monoculture (Table S2).

The results are similar to the molecular basis of the initiation of vascularization and subcutis restoration at 21 days after sample implantation in mice (Figure 4).

Coculturing cells in 2D and especially 3D (in the presence of CaP-coated samples) conditions strongly increased the viability of both blood (from 17–69% to 97% of nonadherent live leukocytes) and stromal (by 7–8%) cells (Tables S5 and S6). At the same time, hBMNCs in close contact with either autologous fibroblast-like cells (Figure 6e) or allogeneic hAMSCs (data not shown) showed more intense alizarin red staining in the cytoplasm and nucleus than freely adherent blood cells (Figure 6d). These results were unusual for this dye, and the increased death of leukocytes adhered to stromal cells has been proposed. MSCs have the capacity for immunomodulatory effects through paracrine signals and/or cell-cell contact [97]. Perhaps, hAMSCs had the opposite influence effect in this context (humoral stimulation of nonadherent hBMNCs and direct contact inhibition).

The number of CD3^+^CD4^+^ hBMNCs increased, and the immunophenotype of these cells changed from proliferative (CD71: Transferrin receptor, a T lymphocyte mitogen [98]; CD25: IL-2 receptor) to activated, as evidenced by the expression of differentiation (CD45RA naïve: CD45RO^+^ activated T lymphocytes and/or T memory cells) and apoptosis (CD95) markers (Table S5). In this regard, the obtained results emphasize T cell phenotype switching induced by hAMSCs alone, as described previously [61], and in combination with CaP material but not the suppression or stimulation of lymphocyte proliferation as described in Reference [72,73], respectively.

It is possible that MSCs altered the profile of naïve and effector T cells to induce a more anti-inflammatory and regenerative phenotype [98]. Indeed, the presence of hBMNCs in hAMSC culture for 14 days limited the expression of stem cell markers (CD73 and CD105; Table S6) and increased the alizarin red staining of mineralization sites (Table 2). Stromal cell motility was diminished (Table 4) and regulated by hBMNCs (Figure 7). CaP-coated specimens strongly enhanced the described changes (Table S6, Table 2 and Table 4; Figure 5) that were defined earlier as hAMSC osteogenic differentiation [27]. This is highly likely because osteoblasts differentiated from MSCs secrete organic bone matrix and induce mineralization [99].

Overall, hBMNCs promoted the invasion of hAMSCs across the model vessel wall (Figure 8), which highlights the significance of a chemotactic gradient induced by hBMNCs for hAMSC recruitment and homing to ectopic sites of inflammation and/or regeneration. Proinflammatory (e.g., TNFα, IL-1, IL-8, RANTES, MCP-1, and MIP-1α) and angiogenic (bFGF, PDGF, VEGF) molecules were secreted in monocultures and mixed cultures (Tables S1, S2 and Table 5); these signals mediate the mobilization and subsequent homing of MSCs [97,100], including in CaP-induced ectopic bone formation [29]. In the presence of CaP-coated specimens, hBMNCs alone and hBMNCs+hAMSCs showed a marked increase in the secretion of biomolecules compared to hAMSCs alone (Tables S1 and S2). Notably, BMNCs can regulate AMSC motility, secretion and osteogenic features as a potential strategy for enhancing reparative regeneration after the ectopic implantation of inorganic biomaterials.

## 5. Conclusions

**Figure 9:**
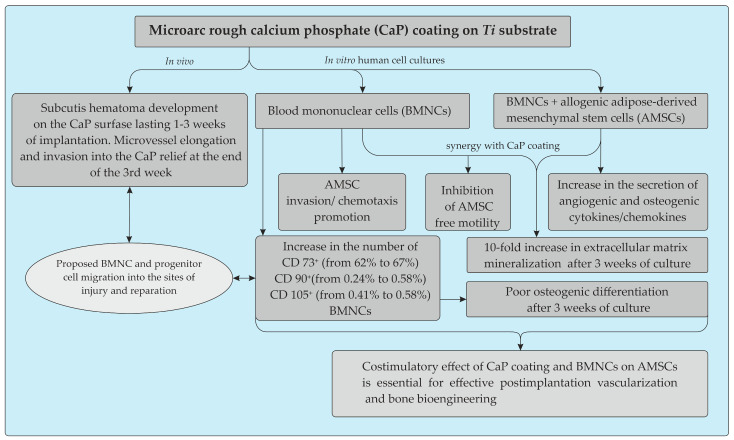
Schematic representation summarizing the in vitro and in vivo effects of the microarc CaP coating on a titanium substrate.

Here, we provide clear in vitro evidence of the costimulatory effect of microarc rough CaP coating and inflammatory BMNCs, mainly CD4^+^ T cells, on the recruitment of AMSCs and their secretion of angiogenic and osteomodulatory molecules and osteogenic features. The main results are presented in Figure 9.

Long-term post-implantation hematoma caused by lateral excursion of the subcutis on the relief CaP surface may play a key role in the vascularization and BMNC invasion required for enhanced tissue regeneration. The infiltration of adipose tissue by T cells peaked earlier than that by macrophages and resulted in the recruitment of additional infiltrating macrophages to enhance inflammation and the reconstruction of soft tissue defects [77]. Our data emphasize the significance of the T cell status for CaP implantation, which is not always successful at inducing subcutaneous vascularization and bone formation without osteogenic cells or bone growth factors [9,12]. The in vitro coregulation of local AMSC motility and invasion, secretion and osteogenic features by CaP coating and emigratory BMNCs may be a model for enhancing ectopic vascularization and bone growth after the subcutaneous implantation of CaP biomaterials.

MSCs have the capacity to home to and integrate into damaged tissue and exert immunomodulatory effects that are regulated by the local inflammatory microenvironment [97]. Such capabilities are essential for the development of suitable cellular therapeutic methods and clinical applications based on MSCs. BM-MSCs are considered more capable of osteogenic differentiation than AMSCs and show superior bone formation [5]. Different strategies to enhance AMSC capacities to match or exceed those of BM-MSCs will improve future clinical applications [66]. Thus, preconditioning and/or co-transplantation of hAMSCs with allogeneic hBMNCs may broaden their clinical potential in applications related to post-implantation tissue repair and bone bioengineering caused by microarc CaP coating.

## Figures and Tables

**Table 1:** Topography parameters of titanium substrates with microarc rough CaP coating, mean (X) ± standard deviation (SD).

First Measurement, *n* = 10, *n_1_* = 100			Second Measurement on Other Samples, *n* = 16, *n_1_* = 48	
*R_a_*, µm	*R_z_*, µm	*S_m_*, µm	*R_a_*, µm	Total Area (S) of Surface Valleys between Spherulites, %
4.15 ± 1.20	15.86 ± 3.69	100.20 ± 11.10	3.14 ± 0.99	46 ± 7

Note: *n*=number of samples tested; *n_1_*=number of measurements.

**Table 2:** In vitro hAMSC and hBMNC osteogenic differentiation with ECM mineralization after 21 days of culture, Me (Q1–Q3), *n* = 3.

Parameters of Bilateral CaP Coating			Biological Parameters		
*Ra*, µm	Thickness, µm	Mass, mg	The Number of the Sites of ECM Mineralization Calculated in 3 Wells	An Average Area of the Mineralization Sites, mm^2^	Total Area of the Stes of ECM Mineralization, mm^2^
(a) hBMNC culture on plastic surface					
-	-	-	2 (0–20)	-*	-*
(b) hAMSC culture on plastic surface (2D control)					
-	-	-	0 *	0	0
(c) hAMSC culture in contact with the CaP-coated titanium substrates					
2.6 (2.3–3.0)	37.5 (35.0–47)	10.5 (9.4–13.8)	0 *	0	0
(d) hAMSC and hBMNC coculture on plastic surface (2D control 1)					
-	-	-	34 ^b^ (25–38)	0.15 ^b^ (0.04–0.43)	9.56 ^b^ (1.33–15.54)
(e) hAMSC and hBMNC coculture on plastic surface in contact with the CaP-coated titanium substrates (3D mixed culture)					
2.7 (2.4–3.0)	40.0 (36.0–47.5)	12.2 (10.9–14.0)	190 ^c,d^ (176–217)	0.34 ^c^ (0.31–0.49)	95.63 ^c,d^ (68.08–102.41)

Note: Significant differences were determined with the Mann–Whitney *U* test: ^b–d^ *P* < 0.05 vs. the corresponding group number; *n* = number of tested wells in each culture plate for each group; total area = average area × number of stained sites in each of 3 wells; *) alizarin red staining of multiple cells and the smallest ECM sites was observed only.

**Table 3:** Monitoring hAMSC division during 7-day culture without or with hBMNCs and/or the rough CaP coating, Me (Q1–Q3), X ± SD, *n* = 3.

Bilateral CaP Coating Parameters			Average Velocity of Cell Division (AVCD), Number of Divisions per Hour Before Monolayer Formation
*Ra*, µm	Thickness, µm	Mass, mg	
(a) hAMSC culture on plastic surface (2D control)			
-	-	-	0.40 ± 0.29 *n_1_* = 18
(b) hAMSC culture in contact with the CaP-coated titanium substrates			
3.5 (2.4–4.3)	52.0 (30.5–56.5)	14.0 (9.0–17.1)	0.46 ± 0.30 *n_1_* = 13
(c) hAMSC and hBMNC coculture on plastic surface (2D control 1)			
-	-	-	0.44 ± 0.31 *n_1_* = 31
(d) hAMSC and hBMNC coculture on plastic surface in contact with the CaP-coated titanium substrates			
3.3 (2.3–4.3)	51.0 (31.5–52.5)	13.7 (9.3–14.7)	0.39 ± 0.29 *n_1_* = 12

**Table 4:** Monitoring hAMSC motility during 7 days of culture without or with hBMNCs and/or the rough CaP coating, Me (Q1; Q3), *n* = 3.

*Ra*, µm	Thickness, µm	Mass, mg	Visualization Fields According to Figure 1	Alteration of hAMSC Count by the End of Observation	Average Alteration Rate (AAR) of hAMSC Number per Hour
(a) hAMSC culture on plastic surface (2D control)					
-	-	-	1	390 (280;420)	2.55 (2.36;2.63)
			2	90 (40;250)	1.0 (0,67;4.55)
			3	0 (0;10)	0 (0;2)
			4	430 (410;470)	2.85 (2.48;3.19)
			5	210 (180;310)	3.65 (2.39;5.14)
			6	10 (0;45)	0.67 (0;0.75)
(b) hAMSC culture in contact with the CaP-coated titanium substrates					
3.5 (2.4–4.3)	52.0 (30.5-56.5)	14.0 (9.0–17.1)	1	368 (310;450)	2.3 (2.3;2.73)
			2	30 (10;180)	0.45 (0.38;2.5)
			3	0 (0;45)	0 (0;0.53)
			4	370 (350;390)	2.44 (2.12;2.74)
			5	60 (38;100)	0.86 (0.54;1.09) ^a^
			6	0 (0;0)	0 (0;0)
(c) hAMSC and hBMNC coculture on plastic surface (2D control 1)					
-	-	-	1	290 (200;295)	1.79 (1.21;2.36)
			2	10 (0;20)	0.37 (0;1.33)
			3	155 (0;155)	0 (0;1.41)
			4	235 (100;250)	1.52 (1.18;1.62) ^a^
			5	130 (35;155)	1.41 (1.09;1.53) ^a^
			6	0 (0;0)	0 (0;0)
(d) hAMSC and hBMNC coculture on plastic surface in contact with the CaP-coated titanium substrates					
3.3 (2.3–4.3)	51.0 (31.5–52.5)	13.7 (9.3–14.7)	1	235 (180–260)	1.58 (0.95–1.6) ^b^
			2	0 (–10; 0)	0 (–0.11; 0) ^b^
			3	11 (–20; 15)	0.11 (–0.17; 0.14)
			4	180 (168;235)	1.12 (1.02; 1.42) ^b^
			5	10 (14;195)	0.14 (–0.08;3.9) ^c^
			6	0 (0;0)	0 (0;0)

Note: The minus sign indicates a decreased number of migratory cells in the visualization field compared with initial observation timepoint. Significant differences were determined with the Mann–Whitney *U* test: (**a**–**c**) *P* < 0.05 vs. the corresponding experimental group number; *n*, number of wells in the plate for each group.

**Table 5:** Significant increases in cytokines, growth factors and chemokines with angiogenic and osteomodulatory potential in 14-day mixed hAMSC + hBMNC cultures.

Cytokines	Increased Concentrations (Table 3) vs. hAMSC Monoculture, Times	Angiogenic Properties	References	MSC, Osteoblast, and Osteoclast Network; Bone Remodeling	References
Inflammatory interleukins and cytokines					
IL-1	1.5	+	[74]	+/-	[33,74]]
IL-1Ra	6	-	[75]	+	[76]
IL-2	2	+	[77]	+	[78]
IL-4	1.5	+	[79]	+	[79]
IL-5	9	?	[80]	?	[81]
IL-6	6	+	[80,82]	+/-	[33]
IL-9	1.3	+	[83]	?	[84]
IL-10	1.8	-	[80,85]	+	[33]
IL-12	1.6	-	[80]	+	[78]
IL-13	6.5	+	[86]	+	[33,78]
IL-17	1.4	+	[82]	+/-	[33]
TNFα	1.3	+/-	[87]	+/-	[33,74]
IFNγ	1.5	-	[80,85]	-	[88]
Growth factors					
G-CSF	-	+	[89]	+	[89]
GM-CSF	-	+	[90]	+	[91]
Angiogenic growth factors					
bFGF	1.4	+	[51]	+	[92]
VEGF	1.3	+		+	[92]
PDGF-BB	1.4	+		+	[93]
Chemokines					
Eotaxin (CCL11)	4	+/-	[94,95]	+	[96]

## Supplementary Materials

The following are available online at https://www.mdpi.com/1996-1944/13/19/4398/s1, Table S1. Secretory activity (pg/mL) of hBMNCs (10^6^ live cells per 1.5 mL) after 2 days in culture without or with the rough CaP coating, Me (Q_1_–Q_3_); Table S2. Secretory activity (pg/mL) of hBMNCs (10^6^ live cells per 1.5 mL) and hAMSCs (5 × 10^4^ viable cells per 1.5 mL) after 14 days of monoculture or coculture without or with the rough CaP coating, Me (Q_1_–Q_3_); Table S3. Immunophenotype and viability of hBMNCs after 2 days of culture without or with the rough CaP coating, Me (Q_1_–Q_3_); Table S4. Immunophenotype of hBMNCs after 3 days of culture without or with the rough CaP coating, Me (Q_1_–Q_3_); Table S5. Immunophenotype and viability of hBMNCs after 14 days of culture without or with hAMSCs and/or the rough CaP coating, Me (Q_1_–Q_3_); Table S6. Immunophenotype and viability of hAMSCs after 14 days of culture without or with hBMNCs and/or the rough CaP coating, Me (Q_1_–Q_3_).

## References

[1] Scott M.A., Levi B., Askarinam A., Nguyen A., Rackohn T., Ting K., Soo C., James A.W. (2012). Brief review of models of ectopic bone formation. Stem Cells Dev..

[2] Tavassoli M. (1984). Hemopoiesis in ectopically implanted bone marrow. Kroc Found. Ser..

[3] Urist M.R., McLean F.C. (1952). Osteogenetic potency and new-bone formation by induction in transplants to the anterior chamber of the eye. J. Bone Joint Surg. Am..

[4] Gamie Z., Tran G.T., Vyzas G., Korres N., Heliotis M., Mantalaris A., Tsiridis E. (2012). Stem cells combined with bone graft substitutes in skeletal tissue engineering. Expert Opin. Biol. Ther..

[5] Im G.-I., Shin Y.-W., Lee K.-B. (2005). Do adipose tissue-derived mesenchymal stem cells have the same osteogenic and chondrogenic potential as bone marrow-derived cells?. Osteoarthr. Cartil..

[6] Khlusov I.A., Karlov A.V., Sharkeev Y.P., Pichugin V.F., Kolobov Y.P., Shashkina G.A., Ivanov M.B., Legostaeva E.V., Sukhikh G.T. (2005). Osteogenic potential of mesenchymal stem cells from bone marrow in situ: Role of physicochemical properties of artificial surfaces. Bull. Exp. Biol. Med..

[7] Matsushima A., Kotobuki N., Tadokoro M., Kawate K., Yajima H., Takakura Y., Ohgushi H. (2009). In vivo osteogenic capability of human mesenchymal cells cultured on hydroxyapatite and on beta-tricalcium phosphate. Artif. Organs.

[8] Seyedjafari E., Soleimani M., Ghaemi N., Shabani I. (2010). Nanohydroxyapatite-coated electrospun poly(l-lactide) nanofibers enhance osteogenic differentiation of stem cells and induce ectopic bone formation. Biomacromolecules.

[9] Yamasaki H., Sakai H. (1992). Osteogenic response to porous hydroxyapatite ceramics under the skin of dogs. Biomaterials.

[10] Barradas A.M.C., Yuan H., van Blitterswijk C.A., Habibovic P. (2011). Osteoinductive biomaterials: Current knowledge of properties, experimental models and biological mechanisms. Eur. Cell Mater..

[11] Humbert P., Brennan M.Á., Davison N., Rosset P., Trichet V., Blanchard F., Layrolle P. (2019). Immune modulation by transplanted calcium phosphate biomaterials and human mesenchymal stromal cells in bone regeneration. Front. Immunol..

[12] Ben-David D., Kizhner T., Livne E., Srouji S. (2010). A tissue-like construct of human bone marrow MSCs composite scaffold support in vivo ectopic bone formation. J Tissue Eng. Regen. Med..

[13] Murr L.E. (2019). Strategies for creating living, additively manufactured, open-cellular metal and alloy implants by promoting osseointegration, osteoinduction and vascularization: An overview. J. Mater. Sci. Technol..

[14] Mansilla E., Marín G.H., Drago H., Sturla F., Salas E., Gardiner C., Bossi S., Lamonega R., Guzmán A., Nuñez A. (2006). Bloodstream cells phenotypically identical to human mesenchymal bone marrow stem cells circulate in large amounts under the influence of acute large skin damage: New evidence for their use in regenerative medicine. Transplant. Proc..

[15] Le Nihouannen D., Saffarzadeh A., Gauthier O., Moreau F., Pilet P., Spaethe R., Layrolle P., Daculsi G. (2008). Bone tissue formation in sheep muscles induced by a biphasic calcium phosphate ceramic and fibrin glue composite. J. Mater. Sci. Mater. Med..

[16] Doherty T.M., Asotra K., Fitzpatrick L.A., Qiao J.-H., Wilkin D.J., Detrano R.C., Dunstan C.R., Shah P.K., Rajavashisth T.B. (2003). Calcification in atherosclerosis: Bone biology and chronic inflammation at the arterial crossroads. Proc. Natl. Acad. Sci. USA.

[17] Zimmerlin L., Park T.S., Zambidis E.T., Donnenberg V.S., Donnenberg A.D. (2013). Mesenchymal stem cell secretome and regenerative therapy after cancer. Biochimie.

[18] Baer P.C., Geiger H. (2012). Adipose-derived mesenchymal stromal/stem cells: Tissue localization, characterization, and heterogeneity. Stem Cells Int..

[19] Ratner B., Lemon J.E., Schoen F.J., Ratner B.D. (2004). Biomaterials Science: An Introduction to Materials in Medicine.

[20] Khlusov I.A., Dekhtyar Y., Sharkeev Y.P., Pichugin V.F., Khlusova M.Y., Polyaka N., Tjulkins F., Vendinya V., Legostaeva E.V., Litvinova L.S. (2018). Nanoscale electrical potential and roughness of a calcium phosphate surface promotes the osteogenic phenotype of stromal cells. Materials (Basel).

[21] Sharkeev Y., Komarova E., Sedelnikova M., Khlusov I.A., Eroshenko A., Litvinova L., Shupletsova V. (2019). Bioactive micro-arc calcium phosphate coatings on nanostructured and ultrafine-grained bioinert metals and alloys. Bioceramics and Biocomposites.

[22] World Medical Association (WMA) Declaration of Helsinki (2009). Ethical principles for medical research involving human subjects. Jahrbuch für Wissenschaft und Ethik.

[23] Zuk P.A., Zhu M., Mizuno H., Huang J., Futrell J.W., Katz A.J., Benhaim P., Lorenz H.P., Hedrick M.H. (2001). Multilineage cells from human adipose tissue: Implications for cell-based therapies. Tissue Eng..

[24] Avdeeva E., Shults E., Rybalova T., Reshetov Y., Porokhova E., Sukhodolo I., Litvinova L., Shupletsova V., Khaziakhmatova O., Khlusov I. (2019). Chelidonic acid and its derivatives from saussurea controversa: Isolation, structural elucidation and influence on the osteogenic differentiation of multipotent mesenchymal stromal cells in vitro. Biomolecules.

[25] Dominici M., Le Blanc K., Mueller I., Slaper-Cortenbach I., Marini F., Krause D., Deans R., Keating A., Prockop D., Horwitz E. (2006). Minimal criteria for defining multipotent mesenchymal stromal cells. The International Society for Cellular Therapy position statement. Cytotherapy.

[26] Bourin P., Bunnell B.A., Casteilla L., Dominici M., Katz A.J., March K.L., Redl H., Rubin J.P., Yoshimura K., Gimble J.M. (2013). Stromal cells from the adipose tissue-derived stromal vascular fraction and culture expanded adipose tissue-derived stromal/stem cells: A joint statement of the International Federation for Adipose Therapeutics and Science (IFATS) and the International Society for Cellular Therapy (ISCT). Cytotherapy.

[27] Litvinova L.S., Shupletsova V.V., Yurova K.A., Khaziakhmatova O.G., Todosenko N.M., Khlusova M.Y., Slepchenko G.B., Cherempey E.G., Sharkeev Y.P., Komarova E.G. (2017). Cell-IQ visualization of motility, cell mass, and osteogenic differentiation of multipotent mesenchymal stromal cells cultured with relief calcium phosphate coating. Dokl. Biochem. Biophys..

[28] Litvinova L.S., Shupletsova V.V., Khaziakhmatova O.G., Yurova K.A., Malashchenko V.V., Melashchenko E.S., Todosenko N.M., Khlusova M.Y., Sharkeev Y.P., Komarova E.G. (2018). Behavioral changes of multipotent mesenchymal stromal cells in contact with synthetic calcium phosphates in vitro. Cell Tissue Biol..

[29] Wang M., Chen F., Wang J., Chen X., Liang J., Yang X., Zhu X., Fan Y., Zhang X. (2018). Calcium phosphate altered the cytokine secretion of macrophages and influenced the homing of mesenchymal stem cells. J. Mater. Chem. B.

[30] Ock S.-A., Baregundi Subbarao R., Lee Y.-M., Lee J.-H., Jeon R.-H., Lee S.-L., Park J.K., Hwang S.-C., Rho G.-J. (2016). Comparison of immunomodulation properties of porcine mesenchymal stromal/stem cells derived from the bone marrow, adipose tissue, and dermal skin tissue. Stem Cells Int..

[31] Lee M.W., Ryu S., Kim D.S., Lee J.W., Sung K.W., Koo H.H., Yoo K.H. (2019). Mesenchymal stem cells in suppression or progression of hematologic malignancy: Current status and challenges. Leukemia.

[32] Khlusov I.A., Shevtsova N.M., Khlusova M.Y., Turksen K. (2013). Detection in vitro and quantitative estimation of artificial microterritories which promote osteogenic differentiation and maturation of stromal stem cells. Stem Cell Niche.

[33] Loi F., Córdova L.A., Pajarinen J., Lin T., Yao Z., Goodman S.B. (2016). Inflammation, fracture and bone repair. Bone.

[34] Curtis A., Wilkinson C. (1997). Topographical control of cells. Biomaterials.

[35] Wang Y., Yu H., Chen C., Zhao Z. (2015). Review of the biocompatibility of micro-arc oxidation coated titanium alloys. Mater. Des..

[36] Gnedenkov S.V., Scharkeev Y.P., Sinebryukhov S.L., Khrisanfova O.A., Legostaeva E.V., Zavidnaya A.G., Puz’ A.V., Khlusov I.A. (2011). Formation and properties of bioactive surface layers on titanium. Inorg. Mater. Appl. Res..

[37] Komarova E.G., Sharkeev Y.P., Sedelnikova M.B., Prosolov K.A., Khlusov I.A., Prymak O., Epple M. (2020). Zn-or Cu-containing CaP-based coatings formed by micro-arc oxidation on titanium and Ti-40Nb alloy: Part I—microstructure, composition and properties. Materials.

[38] Legostaeva E.V., Kulyashova K.S., Komarova E.G., Epple M., Sharkeev Y.P., Khlusov I.A. (2013). Physical, chemical and biological properties of micro-arc deposited calcium phosphate coatings on titanium and zirconium-niobium alloy. Mater. Werkst..

[39] Khlusov I.A., Khlusova M.Y., Zaitsev K.V., Kolokol’tsova T.D., Sharkeev Y.P., Pichugin V.F., Legostaeva E.V., Trofimova I.E., Klimov A.S., Zhdanova A.I. (2011). Pilot in vitro study of the parameters of artificial niche for osteogenic differentiation of human stromal stem cell pool. Bull. Exp. Biol. Med..

[40] Anselme K., Bigerelle M. (2014). On the relation between surface roughness of metallic substrates and adhesion of human primary bone cells: Relation surface roughness/cell adhesion. Scanning.

[41] Zigterman B.G.R., Van den Borre C., Braem A., Mommaerts M.Y. (2019). Titanium surface modifications and their soft-tissue interface on nonkeratinized soft tissues—A systematic review (Review). Biointerphases.

[42] Khlusov I.A., Litvinova L.S., Khlusova M.Y., Yurova K.A. (2018). Concept of hematopoietic and stromal niches for cell-based diagnostics and regenerative medicine (a review). Curr. Pharm. Des..

[43] Yuan H., van Blitterswijk C.A., de Groot K., de Bruijn J.D. (2006). Cross-species comparison of ectopic bone formation in biphasic calcium phosphate (BCP) and hydroxyapatite (HA) scaffolds. Tissue Eng..

[44] Schell H., Duda G.N., Peters A., Tsitsilonis S., Johnson K.A., Schmidt-Bleek K. (2017). The haematoma and its role in bone healing. J. Exp. Orthop..

[45] Mizuno K., Mineo K., Tachibana T., Sumi M., Matsubara T., Hirohata K. (1990). The osteogenetic potential of fracture haematoma. Subperiosteal and intramuscular transplantation of the haematoma. J. Bone Jt. Surg. Br..

[46] Schmidt-Bleek K., Schell H., Lienau J., Schulz N., Hoff P., Pfaff M., Schmidt G., Martin C., Perka C., Buttgereit F. (2014). Initial immune reaction and angiogenesis in bone healing. J. Tissue Eng. Regen. Med..

[47] Xiaozhen D., Shaoxi C., Qunfang Y., Jiahuan J., Xiaoqing Y., Xin X., Qifeng J., Albert Chih-Lueh W., Yi T. (2011). A novel in vitro angiogenesis model based on a microfluidic device. Chin. Sci. Bull..

[48] Lienau J., Schmidt-Bleek K., Peters A., Haschke F., Duda G.N., Perka C., Bail H.J., Schütze N., Jakob F., Schell H. (2009). Differential regulation of blood vessel formation between standard and delayed bone healing. J. Orthop. Res..

[49] Semenza G.L. (2007). Vasculogenesis, angiogenesis, and arteriogenesis: Mechanisms of blood vessel formation and remodeling. J. Cell. Biochem..

[50] Ratajska A., Jankowska-Steifer E., Czarnowska E., Olkowski R., Gula G., Niderla-Bielińska J., Flaht-Zabost A., Jasińska A. (2017). Vasculogenesis and its cellular therapeutic applications. Cells Tissues Organs (Print).

[51] Mouta C., Liaw L., Maciag T. (2003). Angiogenesis: Cellular and molecular aspects of postnatal vessel formation. Handbook of Cell Signaling.

[52] Risau W. (1997). Mechanisms of angiogenesis. Nature.

[53] Tite T., Popa A.-C., Balescu L.M., Bogdan I.M., Pasuk I., Ferreira J.M.F., Stan G.E. (2018). Cationic substitutions in hydroxyapatite: Current status of the derived biofunctional effects and their in vitro interrogation methods. Materials (Basel).

[54] Fennema E.M., Tchang L.A.H., Yuan H., van Blitterswijk C.A., Martin I., Scherberich A., de Boer J. (2018). Ectopic bone formation by aggregated mesenchymal stem cells from bone marrow and adipose tissue: A comparative study. J. Tissue Eng. Regen. Med..

[55] Cvetković V.J., Najdanović J.G., Vukelić-Nikolić M.Đ., Stojanović S., Najman S.J. (2015). Osteogenic potential of in vitro osteo-induced adipose-derived mesenchymal stem cells combined with platelet-rich plasma in an ectopic model. Int. Orthop..

[56] Guerrero J., Pigeot S., Müller J., Schaefer D.J., Martin I., Scherberich A. (2018). Fractionated human adipose tissue as a native biomaterial for the generation of a bone organ by endochondral ossification. Acta Biomater..

[57] Eslaminejad M.B., Nikmahzar A., Taghiyar L., Nadri S., Massumi M. (2006). Murine mesenchymal stem cells isolated by low density primary culture system. Dev. Growth Differ..

[58] Arron J.R., Choi Y. (2000). Bone versus immune system. Nature.

[59] Greenblatt M.B., Shim J.-H. (2013). Osteoimmunology: A brief introduction. Immune Netw..

[60] Könnecke I., Serra A., El Khassawna T., Schlundt C., Schell H., Hauser A., Ellinghaus A., Volk H.-D., Radbruch A., Duda G.N. (2014). T and B cells participate in bone repair by infiltrating the fracture callus in a two-wave fashion. Bone.

[61] Yuan X., Logan T.M., Ma T. (2019). Metabolism in human mesenchymal stromal cells: A missing link between hMSC biomanufacturing and therapy?. Front. Immunol..

[62] Massanella M., Negredo E., Pérez-Álvarez N., Puig J., Ruiz-Hernández R., Bofill M., Clotet B., Blanco J. (2010). CD4 T-cell hyperactivation and susceptibility to cell death determine poor CD4 T-cell recovery during suppressive HAART. AIDS.

[63] Deaglio S., Dwyer K.M., Gao W., Friedman D., Usheva A., Erat A., Chen J.-F., Enjyoji K., Linden J., Oukka M. (2007). Adenosine generation catalyzed by CD39 and CD73 expressed on regulatory T cells mediates immune suppression. J. Exp. Med..

[64] Quast C., Alter C., Ding Z., Borg N., Schrader J. (2017). Adenosine formed by CD73 on T cells inhibits cardiac inflammation and fibrosis and preserves contractile function in transverse aortic constriction–induced heart failure. Circ. Heart Fail..

[65] Choi K.-D., Vodyanik M.A., Togarrati P.P., Suknuntha K., Kumar A., Samarjeet F., Probasco M.D., Tian S., Stewart R., Thomson J.A. (2012). Identification of the hemogenic endothelial progenitor and its direct precursor in human pluripotent stem cell differentiation cultures. Cell Rep..

[66] Liao H.-T. (2014). Osteogenic potential: Comparison between bone marrow and adipose-derived mesenchymal stem cells. World J. Stem Cells.

[67] Brennan M.A., Renaud A., Guilloton F., Mebarki M., Trichet V., Sensebé L., Deschaseaux F., Chevallier N., Layrolle P. (2017). Inferior in vivo osteogenesis and superior angiogeneis of human adipose-derived stem cells compared with bone marrow-derived stem cells cultured in xeno-free conditions: Ectopic bone formation with BM and at stem cells. Stem Cells Transl. Med..

[68] Liu Y., Zhou Y., Feng H., Ma G., Ni Y. (2008). Injectable tissue-engineered bone composed of human adipose-derived stromal cells and platelet-rich plasma. Biomaterials.

[69] Jeon O., Rhie J.W., Kwon I.-K., Kim J.-H., Kim B.-S., Lee S.-H. (2008). In vivo bone formation following transplantation of human adipose–derived stromal cells that are not differentiated osteogenically. Tissue Eng. Part A.

[70] Liu Y., Zhao Y., Zhang X., Chen T., Zhao X., Ma G., Zhou Y. (2013). Flow cytometric cell sorting and in vitro pre-osteoinduction are not requirements for in vivo bone formation by human adipose-derived stromal cells. PLoS ONE.

[71] Mussano F., Genova T., Petrillo S., Roato I., Ferracini R., Munaron L. (2018). Osteogenic differentiation modulates the cytokine, chemokine, and growth factor profile of ASCs and SHED. Int. J. Mol. Sci..

[72] Melief S.M., Zwaginga J.J., Fibbe W.E., Roelofs H. (2013). Adipose tissue-derived multipotent stromal cells have a higher immunomodulatory capacity than their bone marrow-derived counterparts. Stem Cells Transl. Med..

[73] Crop M.J., Baan C.C., Korevaar S.S., Ijzermans J.N.M., Weimar W., Hoogduijn M.J. (2010). Human Adipose tissue-derived mesenchymal stem cells induce explosive T-cell proliferation. Stem Cells Dev..

[74] Ding J., Ghali O., Lencel P., Broux O., Chauveau C., Devedjian J.C., Hardouin P., Magne D. (2009). TNF-alpha and IL-1beta inhibit RUNX2 and collagen expression but increase alkaline phosphatase activity and mineralization in human mesenchymal stem cells. Life Sci..

[75] Coxon A., Bolon B., Estrada J., Kaufman S., Scully S., Rattan A., Duryea D., Hu Y.-L., Rex K., Pacheco E. (2002). Inhibition of interleukin-1 but not tumor necrosis factor suppresses neovascularization in rat models of corneal angiogenesis and adjuvant arthritis. Arthritis Rheum..

[76] Rowland C.R., Glass K.A., Ettyreddy A.R., Gloss C.C., Matthews J.R.L., Huynh N.P.T., Guilak F. (2018). Regulation of decellularized tissue remodeling via scaffold-mediated lentiviral delivery in anatomically-shaped osteochondral constructs. Biomaterials.

[77] Yuan Y., Li H., Liao Y., Feng C. (2019). CD8+ T cells are involved in early inflammation before macrophages in a rat adipose tissue engineering chamber model. J Tissue Eng. Regen. Med..

[78] Yuan Y., Chen X., Zhang L., Wu J., Guo J., Zou D., Chen B., Sun Z., Shen C., Zou J. (2016). The roles of exercise in bone remodeling and in prevention and treatment of osteoporosis. Prog. Biophys. Mol. Biol..

[79] Zheng Z.-W., Chen Y.-H., Wu D.-Y., Wang J.-B., Lv M.-M., Wang X.-S., Sun J., Zhang Z.-Y. (2018). Development of an accurate and proactive immunomodulatory strategy to improve bone substitute material-mediated osteogenesis and angiogenesis. Theranostics.

[80] Naldini A., Pucci A., Bernini C., Carraro F. (2003). Regulation of angiogenesis by Th1- and Th2-type cytokines. Curr. Pharm. Des..

[81] Magnusson L.U., Hagberg Thulin M., Plas P., Olsson A., Damber J.-E., Welén K. (2016). Tasquinimod inhibits prostate cancer growth in bone through alterations in the bone microenvironment: Tasquinimod in bone. Prostate.

[82] Huang Q., Duan L., Qian X., Fan J., Lv Z., Zhang X., Han J., Wu F., Guo M., Hu G. (2016). IL-17 promotes angiogenic factors IL-6, IL-8, and Vegf production via Stat1 in lung adenocarcinoma. Sci. Rep..

[83] He J., Wang L., Zhang C., Shen W., Zhang Y., Liu T., Hu H., Xie X., Luo F. (2019). Interleukin-9 promotes tumorigenesis through augmenting angiogenesis in non-small cell lung cancer. Int. Immunopharmacol..

[84] Bryington M., Mendonça G., Nares S., Cooper L.F. (2014). Osteoblastic and cytokine gene expression of implant-adherent cells in humans. Clin. Oral Implant. Res..

[85] Sokolov D.I., Lvova T.Y., Okorokova L.S., Belyakova K.L., Sheveleva A.R., Stepanova O.I., Mikhailova V.A., Sel’kov S.A. (2017). Effect of cytokines on the formation tube-like structures by endothelial cells in the presence of trophoblast cells. Bull. Exp. Biol. Med..

[86] Fukushi J., Ono M., Morikawa W., Iwamoto Y., Kuwano M. (2000). The activity of soluble VCAM-1 in angiogenesis stimulated by IL-4 and IL-13. J. Immunol..

[87] Koch A.E., Halloran M.M., Haskell C.J., Shah M.R., Polverini P.J. (1995). Angiogenesis mediated by soluble forms of E-selectin and vascular cell adhesion molecule-1. Nature.

[88] Takayanagi H., Ogasawara K., Hida S., Chiba T., Murata S., Sato K., Takaoka A., Yokochi T., Oda H., Tanaka K. (2000). T-cell-mediated regulation of osteoclastogenesis by signalling cross-talk between RANKL and IFN-γ. Nature.

[89] Ishida K., Matsumoto T., Sasaki K., Mifune Y., Tei K., Kubo S., Matsushita T., Takayama K., Akisue T., Tabata Y. (2010). Bone regeneration properties of granulocyte colony-stimulating factor via neovascularization and osteogenesis. Tissue Eng. Part A.

[90] Bajek A., Gurtowska N., Olkowska J., Kazmierski L., Maj M., Drewa T. (2016). Adipose-derived stem cells as a tool in cell-based therapies. Arch. Immunol. Ther. Exp..

[91] Ruef N., Dolder S., Aeberli D., Seitz M., Balani D., Hofstetter W. (2017). Granulocyte-macrophage colony-stimulating factor-dependent CD11c-positive cells differentiate into active osteoclasts. Bone.

[92] Tong X., Chen X., Zhang S., Huang M., Shen X., Xu J., Zou J. (2019). The effect of exercise on the prevention of osteoporosis and bone angiogenesis. Biomed. Res. Int..

[93] De la Riva B., Sánchez E., Hernández A., Reyes R., Tamimi F., López-Cabarcos E., Delgado A., Évora C. (2010). Local controlled release of VEGF and PDGF from a combined brushite–chitosan system enhances bone regeneration. J Control Release.

[94] Xing Y., Tian Y., Kurosawa T., Matsui S., Touma M., Yanai T., Wu Q., Sugimoto K. (2016). CCL11-induced eosinophils inhibit the formation of blood vessels and cause tumor necrosis. Genes Cells.

[95] Park J.Y., Kang Y.W., Choi B.Y., Yang Y.C., Cho B.P., Cho W.G. (2017). CCL11 promotes angiogenic activity by activating the PI3K/Akt pathway in HUVECs. J. Recept. Signal Transduct..

[96] Sohn D.H., Jeong H., Roh J.S., Lee H.-N., Kim E., Koh J.H., Lee S.-G. (2018). Serum CCL11 level is associated with radiographic spinal damage in patients with ankylosing spondylitis. Rheumatol. Int..

[97] Yagi H., Soto-Gutierrez A., Parekkadan B., Kitagawa Y., Tompkins R.G., Kobayashi N., Yarmush M.L. (2010). Mesenchymal stem cells: Mechanisms of immunomodulation and homing. Cell Transplant..

[98] Bi B.Y., Lefebvre A.M., Duś D., Spik G., Mazurier J. (1997). Effect of lactoferrin on proliferation and differentiation of the Jurkat human lymphoblastic T cell line. Arch. Immunol. Ther. Exp. (Warsz.).

[99] Harada S., Rodan G.A. (2003). Control of osteoblast function and regulation of bone mass. Nature.

[100] Ito H. (2011). Chemokines in mesenchymal stem cell therapy for bone repair: A novel concept of recruiting mesenchymal stem cells and the possible cell sources. Mod. Rheumatol..

