# Supplementary material for: Costimulatory Effect of Rough Calcium Phosphate Coating and Blood Mononuclear Cells on Adipose-Derived Mesenchymal Stem Cells In Vitro as a Model of In Vivo Tissue Repair
Source: Materials (Basel). 2020 Oct 2;13(19):4398. doi: 10.3390/ma13194398 (PMC7579197; doi:10.3390/ma13194398)
Supplement: Supplementary file 1 [file materials-13-04398-s001.pdf]

materials

Supplementary Materials

Costimulatory Effect of Rough Calcium Phosphate Coating and Blood Mononuclear Cells on Adipose-Derived Mesenchymal Stem Cells In Vitro as a Model of In Vivo Tissue Repair

Igor A. Khlusov [1,2,3,*], Larisa S. Litvinova [1,*], Valeria V. Shupletsova [1], Olga G. Khaziakhmatova [1], Vladimir V. Malashchenko [1], Kristina A. Yurova [1], Egor O. Shunkin [1], Vasilii V. Krivosheev [1], Ekaterina D. Porokhova [3], Anastasiia E. Sizikova [3], Linara A. Safiullina [3], Elena V. Legostaeva [4], Ekaterina G. Komarova [4] and Yurii P. Sharkeev [4,5]

1 Center for Immunology and Cell Biotechnology, Immanuel Kant Baltic Federal University, 236029 Kaliningrad, Russia; vshupletsova@mail.ru (V.V.S.); hazik36@mail.ru (O.G.K.); vlmalashchenko@kantiana.ru (V.V.M.); kristina_kofanova@mail.ru (K.A.Y.); egor.shunkin@gmail.com (E.O.S.); v_krivosheev@inbox.ru (V.V.K.)
2 Research School of Chemistry and Applied Biomedical Sciences, National Research Tomsk Polytechnic University, 634050 Tomsk, Russia
3 Department of Morphology and General Pathology, Siberian State Medical University, 634050 Tomsk, Russia; porohova_e@mail.ru (E.D.P.); a.e.sizikova@gmail.com (A.E.S.); saflee4505@mail.ru (L.A.S.)
4 Laboratory of Physics of Nanostructured Biocomposites, Institute of Strength Physics and Materials Science SB RAS (ISPMS SB RAS), 634055 Tomsk, Russia; lego@ispms.tsc.ru (E.V.L.); katerina@ispms.ru (E.G.K.); sharkeev@ispms.tsc.ru (Y.P.S.)
5 Research School of High-Energy Physics, National Research Tomsk Polytechnic University, 634050 Tomsk, Russia
* Correspondence: khlusov63@mail.ru (I.A.K.); larisalitvinova@yandex.ru (L.S.L.); Tel.: +8-3822-901-101 (ext. 1823) (I.A.K.); Tel.: +7-4012-595-595 (ext. 6631) (L.S.L.)

Table S1. Secretory activity (pg/mL) of hBMNCs (10^6 live cells per 1.5 mL) after 2 days in culture without or with the rough CaP coating, Me (Q_1-Q_3).

**Bilateral CaP Coating Parameters**			**Inflammatory Interleukins and C ytokines**														
***Ra*, μm**	**Thick-ness, μm**	**Mass mg**	**IL-1β**	**IL-1Ra**	**IL-2**	**IL-4**	**IL-5**	**IL-6**	**IL-7**	**IL-9**	**IL-10**	**IL-12 (p70)**	**IL-13**	**IL-15**	**IL-17**	**TNFα**	**IFNγ**
a) Nutrient medium without cells, n = 3																	
-	-	-	0 (0-0)	0 (0-0)	0 (0-0.01)	0 (0-0)	0 (0-0.01)	0 (0-0)	0 (0-0)	0 (0-0)	0.01 (0-0.01)	0 (0-0)	0 (0-0)	0 (0-0)	0 (0-0)	0 (0-0)	0 (0-0)
b) hBMNC culture on plastic surface (2D control), n = 3																	
0	0	0	10.1^{a} (6.6-22.3)	456^{a} (344-458)	18.2^{a} (12.2-43.2)	6.4^{a} (5.0-8.2)	13.3^{a} (10.0-15.3)	93.4^{a} (59.8-153.2)	22.2^{a} (18.7-23.5)	22.3^{a} (16.2-24.2)	15.5^{a} (14.2-19.6)	13.5^{a} (12.1-15.3)	16.7^{a} (15.2-20.7)	21.1^{a} (9.6-23.0)	49.5^{a} (38.1-60.8)	109^{a} (76-103)	291^{a} (230-310)
c) hBMNC culture on plastic surface in contact with the CaP-coated titanium substrates, n = 6																	
3.0 (2.2-3.5)	36.5 (23-52)	9.4 (5.5-12.9)	484^{b} (73-769)	1562^{b} (998-1673)	79^{b} (77-127)	8.1 (7.0-9.8)	17.4 (14.7-19.3)	5352^{b} (567-10141)	12.9 (8.0-28.1)	79.1^{b} (43.7-94.4)	23.0 (18.6-29.9)	14.4 (8.2-28.8)	20.7 (18.5-23.0)	74.4^{b} (32.1-103.6)	134^{b} (88-311)	163 (96-199)	376 (307-433)

Continuous of **Table S1.**

**Angiogenic Molecules**			**Hematopoietic Growth Factors**		**Chemokines**						
**bFGF**	**VEGF**	**PDGF-BB**	**G-CSF**	**GM-CSF**	**IL-8 (CXCL8)**	**Eotaxin (CCL11)**	**IP-10 (CXCL10)**	**MCP-1 (CCL2)**	**MIP-1α (CCL3)**	**MIP-1β (CCL4)**	**RANTES (CCL5)**
a) Nutrient medium without cells, n=3											
0.91 (0-1.56)	0 (0-0)	0 (0-0)	0 (0-0)	1.29 (0.09-1.92)	0 (0-0)	0 (0-0.01)	0 (0-0.01)	0 (0-0)	0 (0-0)	0 (0-0)	0 (0-0.01)
b) hBMNC culture on plastic surface (2D control), n=3											
30.6^{a} (16.2-31.4)	69.2^{a} (64.6-85.8)	442^{a} (406-609)	23.2^{a} (20.9-32.3)	118^{a} (76-121)	3825^{a} (2799-9247)	17.3^{a} (13.1-18.3)	3092^{a} (2871-3547)	55.6^{a} (35.4-58.9)	8.1^{a} (6.8-9.2)	91.8^{a} (84.5-112.2)	5605^{a} (5495-6100)
c) hBMNC culture on plastic surface in contact with the CaP-coated titanium substrates, n=6											
37.6 (32.9-42.9)	59.8 (33.3-147.3)	329 (269-583)	967^{b} (447-2508)	141^{b} (124-153)	19532^{b} (10293-28968)	23.6 (15.7-30.2)	2935 (1056-5606)	864^{b} (480-1601)	915^{b} (57-1048)	1933^{b} (329-4603)	3665 (3125-4389)

Note: In Tables S2-7: *n*, number of wells tested in each group; $^{a–f}P < 0.05$ *vs* the corresponding group number according to the Mann Whitney *U* test. Duplicate probes for each well were measured.

Table S2. Secretory activity (pg/mL) of hBMNCs (10^6 live cells per 1.5 mL) and hAMSCs (5×10^4 viable cells per 1.5 mL) after 14 days of monoculture or coculture without or with the rough CaP coating, Me (Q_1-Q_3).

Bilateral CaP Coating Parameters			Inflammatory Interleukins and Cytokines														
Ra, µm	Thick-ness, µm	Mass, mg	IL-1β	IL-1Ra	IL-2	IL-4	IL-5	IL-6	IL-7	IL-9	IL-10	IL-12 (p70)	IL-13	IL-15	IL-17	TNFα	IFNγ
a) Nutrient medium without cells, n = 3																	
-	-	-	0.02 (0.01-0.05)	0.05 (0.03-0.80)	0 (0-0.53)	0 (0-0)	0 (0-0)	0 (0-0.35)	0 (0-0)	0 (0-0)	0.01 (0.01-0.23)	0 (0-0)	0 (0-0)	0 (0-0)	0 (0-0)	0 (0-0)	0 (0-0)
hBMNCs compartment																	
b) hBMNC culture on plastic surface (2D control), n = 4																	
0	0	0	0.06 (0-0.24)	193^{a} (134-814)	0 (0-1.14)	0.34^{a} (0.19-0.50)	0 (0-0.35)	25.8^{a} (15.4-70.5)	0 (0-0.1)	2.32^{a} (1.94-17.48)	0.04 (0-2.20)	0.52^{a} (0.23-0.70)	1.55^{a} (1.50-1.79)	0 (0-0)	0 (0-22.1)	4.98^{a} (3.45-9.81)	3.61^{a} (0.09-10.41)
c) hBMNC culture on plastic surface in contact with the CaP-coated titanium substrates, n=12; $^{b}P_T < 0.05$																	
3.1 (2.2-4.0)	48.5 (33.0-50.5)	13.1 (9.6-15.8)	2.09^{b} (1.70-2.55)	993 (607-1567)	1.35 (1.10-1.60)	1.21^{b} (0.79-1.57)	0 (0-0.42)	189^{b} (128-225)	0.59 (0-0.99)	52.1^{b} (42.3-61.4)	10.6^{b} (8.8-14.8)	1.51^{b} (0.86-1.74)	2.76^{b} (2.21-3.14)	14.9^{b} (12.9-17.7)	38.5^{b} (35.1-43.3)	33.1^{b} (29.2-39.4)	31.9^{b} (13.9-50.3)
hAMMSCs compartment																	
d) hAMMSC culture on plastic surface (2D control 1), n = 4																	
0	0	0	4.25^{a,b} (3.37-4.81)	174.3^{a} (141.2-194.9)	7.96^{a,b} (6.86-8.57)	2.42^{a,b} (1.78-3.28)	1.94^{a,b} (1.58-2.69)	1873^{a,b} (1458-2237)	4.75^{a,b} (3.35-6.85)	64.7^{a,b} (54.3-69.7)	27.1^{a,b} (24.0-29.3)	70.0^{a,b} (56.4-77.2)	4.88^{a,b} (4.12-5.74)	31.6^{a,b} (28.2-35.9)	59.7^{a,b} (55.0-62.7)	80.6^{a,b} (77.5-94.8)	128^{a,b} (83-148)
e) hAMMSC culture on plastic surface in contact with the CaP-coated titanium substrates, n=12; $^{d}P_T < 0.05$																	
3.0 (2.1-4.1)	48.5 (33.0-50.5)	14.1 (9.9-15.8)	0.57^{c,d} (0.32-0.96)	46.1^{c,d} (24.2-57.6)	0.69^{c,d} (0.24-0.89)	0.57^{d} (0.35-1.08)	0^{d} (0-0.14)	1042^{c,d} (587-1097)	3.25^{c} (2.90-3.95)	5.39^{c,d} (4.92-6.72)	10.70^{d} (6.76-13.12)	29.7^{c,d} (18.1-32.7)	0.75^{d} (0-2.18)	10.0^{d} (4.7-14.4)	1.05^{c,d} (0-2.01)	21.5^{c,d} (12.0-26.2)	47.6^{d} (17.1-76.9)
Mixed hAMMSCs+hBMNCs compartment																	
f) hAMMSC and hBMNC coculture on plastic surface (2D control 2), n = 4; $^{b}P_T<0.05$																	
0	0	0	6.22^{d} (4.92-7.58)	1130^{d} (1087-1199)	14.6^{d} (13.0-16.9)	3.90 (3.34-3.98)	18.1^{d} (16.6-19.1)	7547^{d} (4603-10491)	3.90 (3.36-4.47)	87.2^{d} (85.0-91.8)	48.8^{d} (43.1-52.4)	113^{d} (110-118)	31.1^{d} (29.1-31.4)	39.0 (38.0-39.2)	84.2^{d} (82.8-88.8)	105^{d} (101-114)	188 (123-217)
g) hAMMSC and hBMNC coculture on plastic surface in contact with the CaP-coated titanium substrates, n=12; $^{c}P_T < 0.05$; $^{e}P_T < 0.05$																	
3.2 (2.3-4.1)	48.0 (30.0-58.0)	13.8 (9.1-17.1)	7.46 (6.42-8.72)	1378^{f} (1261-1440)	17.3 (14.6-18.5)	4.18 (3.60-4.66)	16.0 (15.2-23.4)	2243^{f} (1649-4966)	5.35 (4.54-6.06)	90.8 (83.8-97.7)	54.9 (45.6-58.5)	120 (111-125)	32.2 (24.5-33.5)	40.3 (37.6-41.1)	106^{f} (100-115)	108 (101-123)	203 (152-243)

Continuous of **Table S2.**

Angiogenic molecules			Hematopoietic Growth Factors		Chemokines						
**bFGF**	**VEGF**	**PDGF-BB**	**G-CSF**	**GM-CSF**	**IL-8 (CXCL8)**	**Eotaxin (CCL11)**	**IP-10 (CXCL10)**	**MCP-1 (CCL2)**	**MIP-1α (CCL3)**	**MIP-1β (CCL4)**	**RANTES (CCL5)**
a) Nutrient medium without cells, n = 3											
0.91 (0-1.56)	0 (0-0)	0 (0-0)	0 (0-0)	1.29 (0.09-1.92)	0 (0-0)	0 (0-0)	0 (0-0)	0 (0-0)	0 (0-0)	0 (0-0)	0 (0-0)
hBMNCs compartment											
b) hBMNC culture on plastic surface (2D control), n = 5											
1.05 (0-2.14)	0 (0-0.47)	19.54[a] (17.17-35.16)	0 (0-3.74)	1.59 (0.49-16.93)	28.7[a] (23.50-68.78)	2.23[a] (0.69-3.18)	694[a] (178-2199)	4.66 (0-17.71)	3.06[a] (1.15-6.24)	4.37[a] (4.07-26.0)	18.4[a] (4.35-45.5)
c) hBMNC culture on plastic surface in contact with the CaP-coated titanium substrates, n = 3; $^{b}P_T < 0.05$											
14.7[b] (11.9-26.0)	3.77[b] (3.16-4.73)	155[b] (147-163)	26.6[b] (20.6-28.4)	49.3[b] (41.6-56.4)	2148[b] (1599-2992)	6.20[b] (4.85-6.56)	4874[b] (4772-5648)	550[b] (248-586)	35.3[b] (31.2-39.1)	494[b] (449-543)	116[b] (111-193)
hAMMSCs compartment											
d) hAMMSC culture on plastic surface (2D control 1), n = 4											
19.0[a,b] (15.0-21.2)	1814[a,b] (1738-1945)	10.9[a] (10.3-12.6)	2540[a,b] (2477-2697)	43.8[a,b] (35.5-51.3)	1372[a,b] (1263-1585)	44.1[a,b] (41.4-45.2)	5361[a,b] (5123-6967)	499[a,b] (487-537)	89.7[a,b] (83.8-113)	500[a,b] (416-1173)	569[a,b] (414-1112)
e) hAMMSC culture on plastic surface in contact with the CaP-coated titanium substrates, n = 3; $^{d}P_T < 0.05$											
6.39[c,d] (4.67-7.27)	422[c,d] (324-441)	1.28[c,d] (0.42-1.83)	14.6[d] (8.2-19.8)	7.87[c,d] (3.71-12.7)	218[c,d] (134-266)	7.75[d] (6.01-8.29)	146[c,d] (140-180)	251[d] (154-285)	0.36[c,d] (0.30-0.39)	0[c,d] (0-0.18)	57.5[c,d] (40.0-66.2)
Mixed hAMMSCs+hBMNCs compartment											
f) hAMMSC and hBMNC coculture on plastic surface (2D control 2), n = 4; $^{b}P_T < 0.05$											
26.2[d] (21.8-27.3)	2372[d] (2225-2539)	15.4 (12.6-16.9)	3550[d] (3535-3550)	96.7[d] (91.1-99.3)	2021 (1574-2552)	177[d] (171-181)	5985 (5594-7403)	480 (471-492)	59.9[d] (54.7-60.6)	588 (545-693)	470 (430-511)
g) hAMMSC and hBMNC coculture on plastic surface in contact with the CaP-coated titanium substrates, n = 3; $^{e}P_T < 0.05$											
30.7[f] (24.6-33.8)	2292[c] (1996-2720)	17.9[c,f] (17.2-18.7)	3550[c] (3550-3550)	220[f] (161-284)	1701 (1310-2337)	206[c] (147-230)	5682 (5337-7304)	499 (456-515)	75.5[c,f] (72.4-85.8)	557 (446-802)	533[c] (407-648)

Note: $^{a\text{-}g}P_T$ <0.05 *vs* the corresponding group according to the Wilcoxon *T* test.

Table S3. Immunophenotype and viability of hBMNCs after 2 days of culture without or with the rough CaP coating, Me (Q_1-Q_3).

Bilateral CaP Coating Parameters			Live or Dead Cells, %			% $CD45^+CD3^+$ Cells Expressing Specific Membrane Markers							
Ra, µm	Thickness, µm	Mass, mg	Viable cells	Apoptosis	Necrosis	CD3	CD4	CD8	CD71	CD95	CD25	CD45RO	CD45RA
a) hBMNC culture on plastic surface (2D control), *n* = 4													
-	-	-	87 (85-88)	2 (1-2)	11 (10-13)	98.77 (98.55-98.96)	67.18 (65.46-68.28)	21.82 (20.54-23.38)	2.51 (1.94-3.60)	15.35 (14.34-16.58)	9.00 (8.75-9.92)	34.83 (33.56-36.13)	55.35 (54.57-55.69)
b) hBMNC culture on plastic surface in contact with the CaP-coated titanium substrates, *n* = 6													
3.0 (2.2-3.5)	36.5 (23.0-52.0)	9.4 (5.5-12.9)	70 (68-71)	2.5 (2.4-3.9)	27[a] (25-28)	98.58 (98.45-98.70)	67.77 (67.29-68.37)	21.11 (20.60-21.53)	4.08 (3.68-4.37)	14.11 (13.53-16.18)	8.55 (8.21-10.14)	36.72 (35.21-38.43)	56.33 (55.62-59.03)

Table S4. Immunophenotype of hBMNCs after 3 days of culture without or with the rough CaP coating, Me (Q_1-Q_3).

Bilateral CaP Coating Parameters			Stromal Cell Markers, % of Cells			Hematopoietic Cell Markers, % of Cells	
Ra, µm	Thickness, µm	Mass, mg	CD73	CD90	CD105	[CD45,34,14,20]	CD45CD14
a) hBMNC culture on plastic surface (2D control), *n* = 3							
-	-	-	62.09 (61.85-62.24)	0.24 (0.15-0.37)	0.41 (0.30-0.70)	66.48 (65.30-67.24)	9.54 (7.63-10.12)
b) hBMNC culture on plastic surface in contact with the CaP-coated titanium substrates, *n* = 3							
3.9 (2.5-4.5)	49.0 (35.0-60.0)	14.5 (10.3-16.4)	67.10[a] (64.70-70.20)	0.58[a] (0.47-1.07)	0.58[a] (0.50-0.85)	70.9[a] (68.60-72.0)	10.17 (9.70-11.98)

Table S5. Immunophenotype and viability of hBMNCs after 14 days of culture without or with hAMSCs and/or the rough CaP coating, Me (Q_1-Q_3).

**Bilateral CaP Coating Parameters**			**Live or Dead Cells, %**			**% $CD45^+CD3^+$ Cells Expressing Specific Membrane Markers**							
***Ra*, µm**	**Thickness, µm**	**Mass, mg**	**Viable cells**	**Apoptosis**	**Necrosis**	**CD3**	**CD4**	**CD8**	**CD71**	**CD95**	**CD25**	**CD45RO**	**CD45RA**
a) hBMNC culture on plastic surface (2D control), *n* = 3													
-	-	-	69 (55-82)	14 (9-14)	17 (9-31)	99.05 (96.99-99.14)	36.12 (35.74-47.01)	22.69 (17.21-27.18)	68.74 (65.95-80.98)	41.76 (35.71-51.74)	87.11 (81.82-89.47)	80.52 (63.16-86.36)	93.18 (81.51-99.97)
b) hBMNC culture on plastic surface in contact with the CaP-coated titanium substrates, *n* = 3													
3.4 (2.6-4.4)	53 (30-55)	13.4 (9.2-15.2)	17[a] (15-38)	13 (8-15)	69[a] (50-75)	99.57 (99.51-99.85)	34.39 (30.35-36.72)	15.30 (14.47-22.34)	76.57 (71.24-78.43)	38.19 (36.07.-42.30)	92.12 (85.67-96.60)	78.18 (78.07-92.20)	87.27 (86.42-97.8)
c) hAMSC and hBMNC coculture on plastic surface (2D control 1), *n* = 6													
-	-	-	97[a] (92-98)	0[a] (0-2)	3[a] (2-8)	99.45 (99.03-99.70)	69.62[a] (65.69-77.67)	23.22 (17.29-28.14)	27.45[a] (20.02-33.46)	59.46 (40.78-72.43)	65.12[a] (59.22-66.27)	92.03[a] (90.86-94.33)	99.94 (99.82-99.96)
d) hAMSC and hBMNC coculture on plastic surface in contact with the CaP-coated titanium substrates, *n* = 3													
3.3 (2.3-4.3)	51.0 (31.5-52.5)	13.7 (9.3-14.7)	97[b] (97-97)	1[b] (1-1)	2[b] (2-2)	99.18 (98.93-99.75)	76.0[b] (66.83-79.94)	19.49 (17.32-27.95)	28.48[b] (12.02-46.85)	68.12[b] (45.99-69.0)	68.78[b] (61.39-70.91)	93.68 (91.26-94.06)	99.96[b] (99.93-99.97)

Table S6. Immunophenotype and viability of hAMSCs after 14 days of culture without or with hBMNCs and/or the rough CaP coating, Me (Q_1-Q_3).

Bilateral CaP Coating Parameters			Live or Dead Cells, %			Stromal Cell Markers, %			Hematopoietic Cell Markers, %
Ra, µm	Thickness, µm	Mass, mg	Viable cells	Apoptosis	Necrosis	CD73	CD90	CD105	[CD45,34,14,20]
a) hAMSC culture on plastic surface (2D control), *n* = 3									
-	-	-	83 (82-84)	10 (8-11)	9 (5-9)	96.55 (96.34-97.17)	91.28 (78.22-93.32)	92.94 (92.89-94.32)	1.21 (1.10-1.34)
b) hAMSC culture on plastic surface in contact with the CaP-coated titanium substrates, *n*=3									
3.5 (2.4-4.3)	52.0 (30.5-56.5)	14.0 (9.0-17.1)	90[a] (90-91)	6[a] (6-7)	3[a] (2-3)	92.41[a] (86.50-94.42)	87.70 (79.43-91.76)	87.48[a] (81.73-88.63)	1.32 (1.26-1.37)
c) hAMSC and hBMNC coculture on plastic surface (2D control 1), *n* = 6									
-	-	-	84 (74-87)	6 (5-9)	10 (8-16)	93.33[a] (89.63-95.76)	78.97 (78.30-95.76)	90.83[a] (84.72-92.29)	1.26 (1.02-1.63)
d) hAMSC and hBMNC coculture on plastic surface in contact with the CaP-coated titanium substrates, *n* = 3									
3.3 (2.3-4.3)	51.0 (31.5-52.5)	13.7 (9.3-14.7)	92[c] (90-96)	5 (3-6)	2[c] (1-4)	88.52[c] (79.25-89.50)	77.73 (63.88-79.73)	88.52 (76.47-89.32)	1.01[b] (0.89-1.03)
